# Carbon Nanotube Reinforced Structural Composite Supercapacitor

**DOI:** 10.1038/s41598-018-34963-x

**Published:** 2018-12-05

**Authors:** Nitin Muralidharan, Eti Teblum, Andrew S. Westover, Deanna Schauben, Anat Itzhak, Merav Muallem, Gilbert D. Nessim, Cary L. Pint

**Affiliations:** 1 Department of Mechanical Engineering, Vanderbilt University, Nashville, TN 37235 USA; 2 Interdisciplinary Materials Science Program, Vanderbilt University, Nashville, TN 37235 USA; 3 Department of Chemistry, Bar Ilan Institute for Nanotechnology and Advanced Materials (BINA), Bar Ilan University, 52900 Ramat Gan, Israel

## Abstract

Carbon nanotubes exhibit mechanical properties ideally suited for reinforced structural composites and surface area and conductivity attractive for electrochemical capacitors. Here we demonstrate the multifunctional synergy between these properties in a composite material exhibiting simultaneous mechanical and energy storage properties. This involves a reinforcing electrode developed using dense, aligned carbon nanotubes grown on stainless steel mesh that is layered in an ion conducting epoxy electrolyte matrix with Kevlar or fiberglass mats. The resulting energy storage composites exhibit elastic modulus over 5 GPa, mechanical strength greater than 85 MPa, and energy density up to 3 mWh/kg for the total combined system including electrodes, current collector, Kevlar or fiberglass, and electrolyte matrix. Furthermore, findings from *in-situ* mechano-electro-chemical tests indicate simultaneous mechanical and electrochemical functionality with invariant and stable supercapacitor performance maintained throughout the elastic regime.

## Introduction

Carbon fiber reinforced structural composites are a benchmark material for structural applications due to high strength to weight ratio and controllable form factor^1–4^. However, the extraordinary stiffness, strength, and conductivity of carbon nanotubes (CNTs) have made them the obvious candidate for next-generation reinforced composite materials^5,6^. In parallel to this, nanoscale activated carbons have provided the benchmark for high power electrochemical supercapacitors due to their conductivity, high surface area, and electrochemical stability^7,8^, and CNTs and graphene based materials similarly have emerged as viable candidates in this application for next-generation high power energy storage^9–22^. Most carbon based supercapacitive materials with extraordinarily high surface areas are not compatible with a structurally reinforced composite design with the exception of CNTs which enable non-faradaic energy storage capabilities while simultaneously providing high surface area benefits. These concepts lay the framework for the emergence of a new class of CNT-based energy storing materials, which can be developed using traditional composite processing routes, exhibit mechanical properties rivaling commercial composites, and have the built-in capability to be energized and environmentally stable. This addresses a growing technological need for “on-demand” energy in systems that exhibit little tolerance for external payload weight, especially for high power applications where large battery footprints are necessary.

Early considerations of structural energy storage composites originated with the premise that commercially packaged lithium-ion batteries can be embedded into the wings of unmanned autonomous vehicles (UAV), the doors of cars, and the walls of marine vessels^23–25^. The challenge of this approach is that the strength of these architectures is bottlenecked by the battery packaging. Recently, a more elegant approach has emerged where the composite materials themselves can simultaneously act as both a structural material and energy storage material, presumably with light or no external packaging. Whereas initial efforts in this area explore structural batteries^26,27^, the requirement for structural materials to withstand environmental exposure and maintain lifetime synergistic with structural components in systems (tens of years) is not compatible with the state-of-the-art durability reported for batteries. In this manner, electric double layer capacitors (EDLCs) emerge as an attractive direction since the non-Faradaic storage mechanism of these devices leads to stability of commercial systems often quoted beyond a million cycles which is of great interest for materials integrated into structural components in expensive systems such as buildings or vehicles^9,28–33^.

In the past few years, a few limited approaches have been demonstrated to develop EDLC devices with structural integrity. Most notably, recent work by the Greenhalgh group combined carbon fiber current collectors with high surface area aerogel electrodes with poly-ethylene glycol diglycidal ether (PEDGE) – ionic liquid (IL) electrolytes^33^. This route yields a composite device performance with energy density up to 0.1 mWh/kg, power density up to 3.8 mW/kg, ultimate tensile strength of 8.71 MPa, and a modulus of 0.9 GPa. However, this design is limited to extremely low operating voltages near 0.4 V and similar to other related studies in this area displays mechanical and electrochemical properties that are assessed independent of each other^26,28,30,32–38^. Recently, Senokos *et al*.^39^, developed a CNT-Fiber based supercapacitor with energy densities as high as 11.4 Wh/kg and power densities of 46 kW/kg based on active mass of the electrodes highlighting the potential of carbon based materials, especially CNTs in structural energy storage. Also, the recent work of Guan Wu *et al*.^40^, has established the benefits of employing aligned carbon nanotubes in supercapacitor application in obtaining high energy and power densities. However, despite some progress until now, key challenges still exist between the conceptual idea of a structural energy storage material and the practical design of such a material. Specifically, a structural material must exhibit stability in diverse environmental conditions without packaging, must exhibit energy storage *while exposed* to mechanical stresses^41^, and must be capable of energy density, discharge voltages, and structural integrity all simultaneously relevant to application markets of existing products^34^. Moreover, unlike conventional energy storage devices which are generally packaged under compression, mechanical integrity across electrode/current collector and electrode/electrolyte interfaces is a basic design scheme for a composite material which has not been an element of any prior studies on structural energy storage materials. Apart from selection of the ideal structural material, interface engineered architectures incorporating such ideal structural materials are a critical design component of such multifunctional systems. Until now, even the basic premise of multifunctional performance assessed by simultaneous testing of electrochemical and mechanical performance of such materials has remained elusive^9,41,42^ despite this being the most critical metric of a material that can simultaneously store and release charge and maintain mechanical integrity.

In this report, we demonstrate the ability to synthesize high density networks of aligned CNTs directly on the surface of stainless steel mesh materials to enable a reinforcing structural and energy storage interface in a multifunctional composite material. This design involves alternating CNT-reinforced electrode interfaces layered with either Kevlar or fiberglass mesh in an ion-conducting diglycl based epoxy-ionic liquid matrix. The direct growth of CNTs to produce the reinforcing interface overcomes challenges of achieving CNT-matrix reinforcement and leveraging the high surface area of CNTs for electrically accessible EDLC charge storage^43,44^. Our findings demonstrate simultaneous mechanical and electrochemical performance with total system energy density up to 3 mWh/kg with power densities up to 1 W/kg, a modulus over 5 GPa, and ultimate tensile strength above 85 MPa. Our testing demonstrates the energy storage performance is maintained in this device configuration throughout the elastic regime of the devices, with steady degradation measured in the plastic regime. Our work lays an important framework for the design and critical assessment approaches for a future class of structurally reinforced energy storage materials.

**Figure 1:**
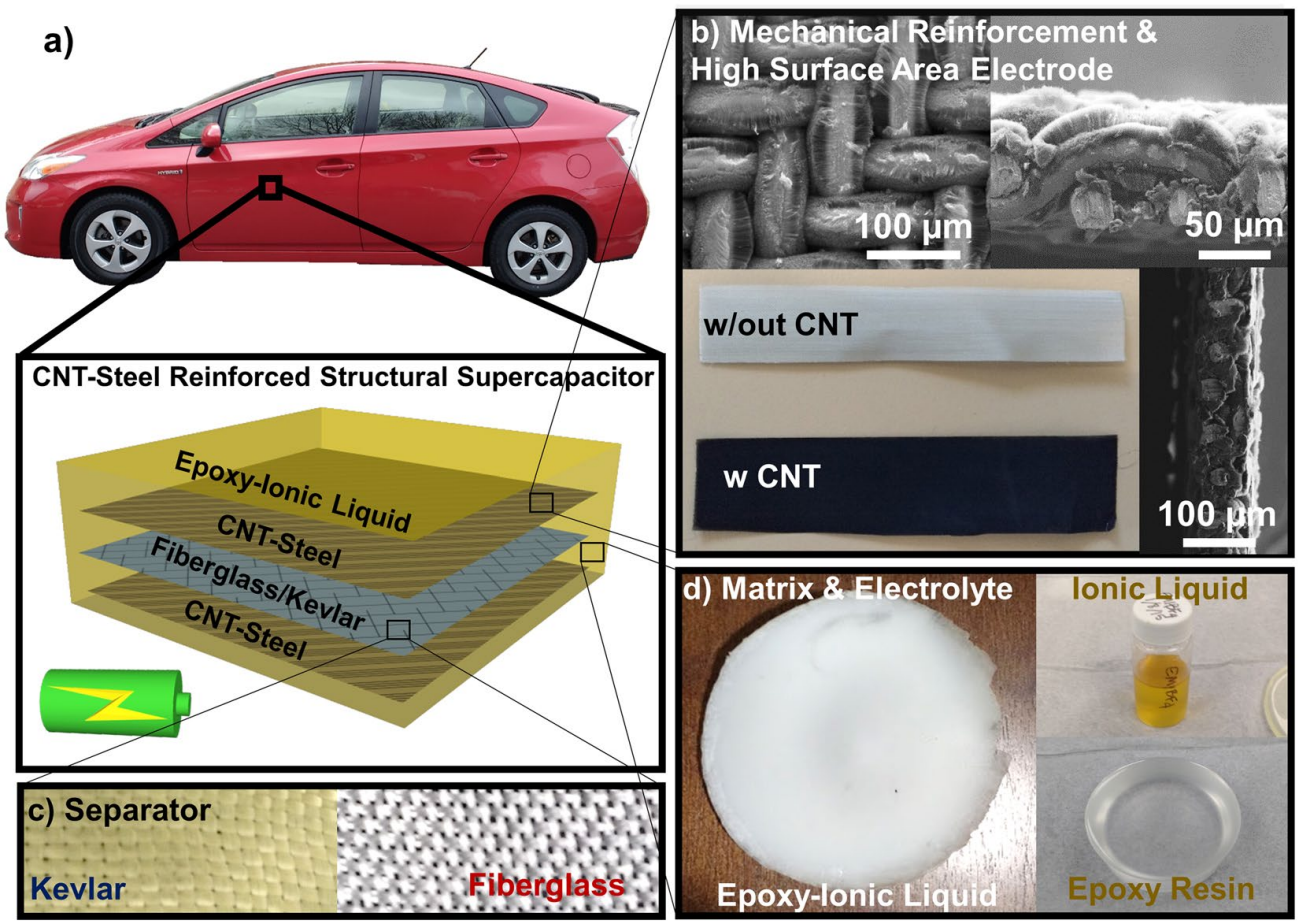
Design of CNT reinforced structural supercapacitor. (**a**) Schematic showing the structural supercapacitor with the vision of a reinforced composite material that could be used in a vehicle chassis. (**b**) SEM and optical images of a CNT-steel mesh. d) Photograph of fiberglass and Kevlar separators. (**c**) Photographs of the epoxy-IL electrolyte on the left and the individual components on the right.

Figure 1 illustrates the make-up of the structural composite with a photograph and schematic of the structural supercapacitor depicted in Fig. 1a. For the electrode materials, CNT were grown on stainless steel meshes *via* chemical vapor deposition (CVD)^45–48^. Three types of stainless steel mesh (316 stainless steel (SS) 200 mesh, 316 SS 400 mesh, and 304 SS 200 mesh and several different growth temperatures (740–790 °C) were analyzed to optimize CNT growth (Fig. S1). CNTs grown on the 316 SS 400 mesh at 790 °C and the growth on the 304 SS 200 mesh at 770 °C were adopted for our device studies based on the highest CNT growth density (see SI, Figs S4 and S5 and Table S6). The average mass loading of the CNTs on the stainless-steel mesh was between 0.30–0.40 mg/cm^2^ (Fig. S5), and CNTs were measured to have thickness between 15–80 µm. Additional microstructural characterization (SEM, TEM) and Raman spectra of the CNTs grown on stainless steel mesh is provided in the Supporting Information Figs S2 and S3. Figure 1b shows three bird’s eye and cross-sectional SEM images of the best CNT growth as well as a photograph of the CNT coated SS steel in comparison with the uncoated SS. Here, CNTs completely coated the steel mesh with lengths ranging from 15–30 µm. Also, the CNT growth incurs uniform color change of the mesh from silver to metallic black. Figure 1c shows photographs of the epoxy-IL composite used for the electrolyte in comparison to photographs of the plain IL and pure epoxy material. These pictures indicate the color of the epoxy with the IL incorporated leads to an opaque white material indicative of light scattering from the boundaries within the bi-phasic material. It should be noted that in this approach the CNTs will not form aggregates in the polymer electrolyte matrix since they are tethered to the stainless-steel substrate from which they are grown. Finally Fig. 1d shows photographs of the two different types of insulating layering materials used in the multifunctional composites, namely fiberglass and Kevlar.

**Figure 2:**
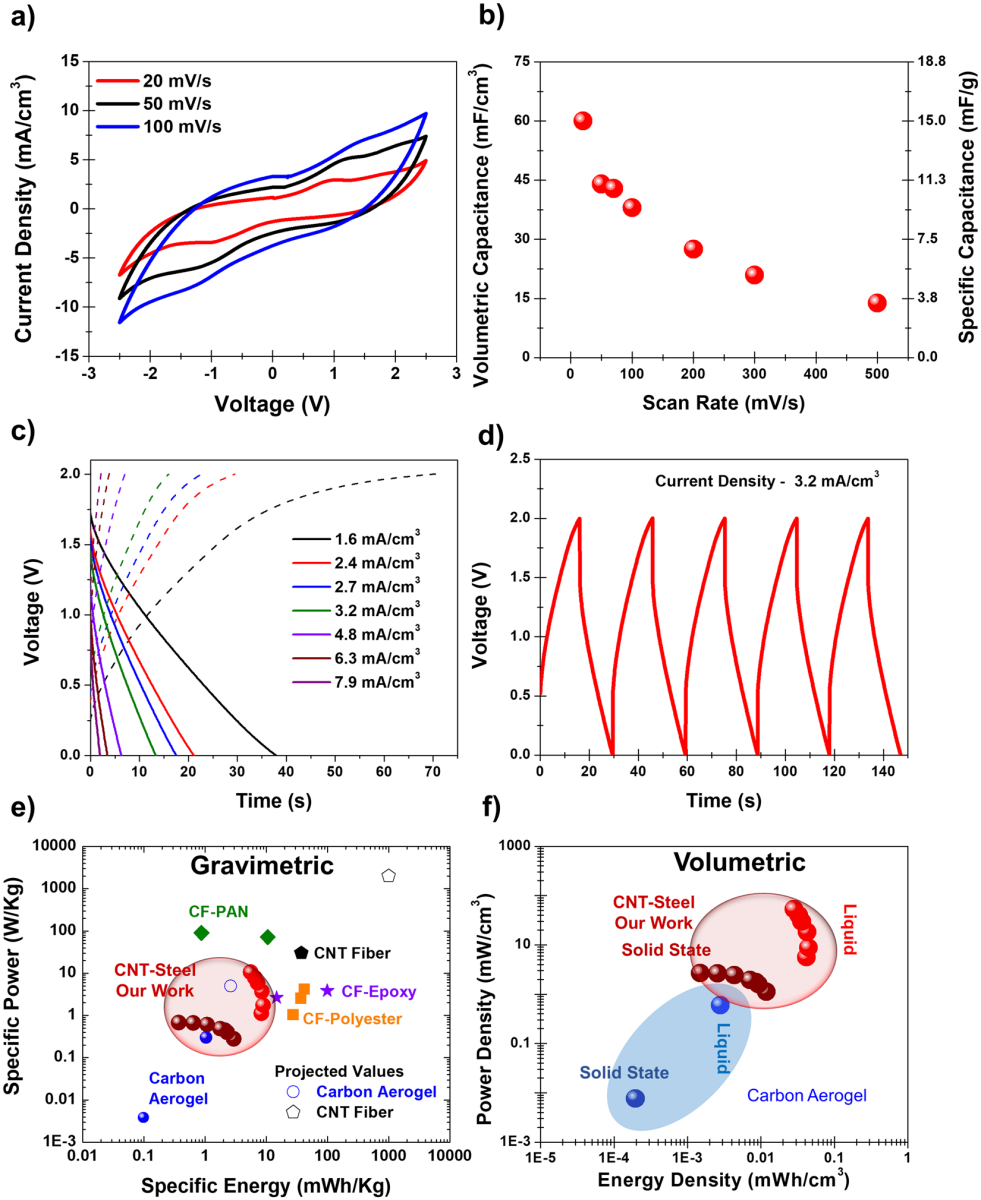
Electrochemical characterization of the structural supercapacitor. (**a**) CV curves from 20 mV/s to 100 mV/s for a composite structural supercapacitor with a Kevlar separator. (**b**) Volumetric and specific capacitance calculated from CV curves for the composite supercapacitors. (**c**) Charge/discharge curves for charging currents ranging from 1.5–8 mA/cm^3^. (**d**) Five consecutive charge-discharge measurements for the composite at 3.2 mA/cm^3^. (**e**) Ragone plot showing specific energy and power density of CNT-Steel mesh performance with a Kevlar separator and a 100% IL electrolyte and an Epoxy-IL electrolyte compared to the liquid/solid state performance of the best structural supercapacitor composites in literature. (**f**) Ragone plot for the same data in the context of volumetric performance compared to carbon aerogels.

Electrochemical testing of CNTs grown on 316 SS 400 meshes in IL electrolytes demonstrated capacitance of ~120 mF/cm^3^, and significantly improved capacitance over SS meshes with no CNTs (Fig. S7). For composite design, electrochemical tests were performed on insulating layering materials including polyethylene, fiberglass, and Kevlar (Fig. S8), and findings that Kevlar and fiberglass exhibit slightly increased resistance and the fiberglass separator exhibits electrochemical window around 2 V. Following this, structural composites were assembled with the CNTs grown on the 316 SS 400 mesh electrodes, a Kevlar separator, and a 45–55 Epoxy-IL electrolyte and electrochemical performance was assessed (Fig. 2). The electrolyte is a critical component of any multifunctional energy storage device and based on our previous work^41^, non-hygroscopic epoxy based electrolytes containing ionic liquid were chosen owing to the good environmental stability and feasibility of effective integration with the high surface area CNTs. Based on measurements reported in our previous work, the ionic conductivity for 55% ionic liquid epoxy mixture was ~0.1 mS/cm^−1^ ^41^. Cyclic voltammetry (CV) curves reflect an electrochemical window of ~2.5 V and maximum capacitance at slow rates of 60 mF/cm^3^ (16 mF/g) and a capacitance of 15 mF/cm^3^ (3 mF/g) at fast rates of 500 mV/s. The electrochemical performance in ionic liquid electrolyte for the stainless steel meshes with and without the CNT growth is provided in the Supporting Information (Fig. S9) for comparison. The volumetric capacitance of the CNT grown stainless steel meshes was 90 mF/cm^3^ and stainless steel meshes without CNTs was 20 mF/cm^3^ at a scan rate 20 mV/s. This is comparable to the best carbon aerogel based devices in literature that have a similar capacitance of about 35 mF/cm^3^ (74 mF/g), but that exhibit much lower energy density and thus have limited practical applicability due to their electrochemical window below 0.4 V. Galvanostatic charge-discharge curves for multiple currents ranging from 1.5 mA/cm^3^ to 8 mA/cm^3^ (Fig. 2c,d) indicate stable performance of the supercapacitive device. It should be noted that the voltage drop or the DCIR (Direct Current Internal Resistance) increases as the distance between the two electrodes of the device increases. Though the electrolyte by itself can function as a separation between the two electrodes as shown in our previous work^41,42,49^, improved mechanical strength requirements of such multifunctional devices warrants the use of mechanically robust separator materials such as Kevlar/fiber glass which increases the separation between the two electrodes resulting in increased voltage drop. Nevertheless, for such mechanical performance dependent systems, reliable mechanical behavior without compromising energy storage functions by preventing shorting in the device can be achieved through the use of such separator materials. The discharge curves form the basis of volumetric energy and power calculations represented in Fig. 2e,f. This is compared with performance of several other structural devices in literature with liquid and solid-state electrolytes. Specifically, we have also included projected values of energy and power densities based on an assumed increase in voltage window for the previously reported carbon aerogel devices^33^ and assuming only mass of the active components for the CNT Fiber based devices from the work of Senokos *et al*.^39^. Though active mass based performance assessment is an important metric for supercapacitive devices, projected values based on full composite mass would be significantly lower owing to the addition of several inactive design components which become essential in full structural device architectures. For our device, we calculate energy density up to 3 mWh/kg (10 µWh/cm^3^) and power density of 1 W/kg (70 mW/cm^3^) by direct integration of discharge voltage profiles, yielding the exact energy and power performance. This compares to equivalent devices having liquid electrolytes with 10 mWh/kg (30 µWh/cm^3^) energy and 10 W/kg (700 mW/cm^3^) power densities (Fig. S10). Notably, our devices exhibit comparable performance in both power and energy densities using both specific and volumetric assessments in comparison to other state-of-the-art reports in literature and the corresponding data are summarized in Table S11 ^32,33,35,39,50^.

**Figure 3:**
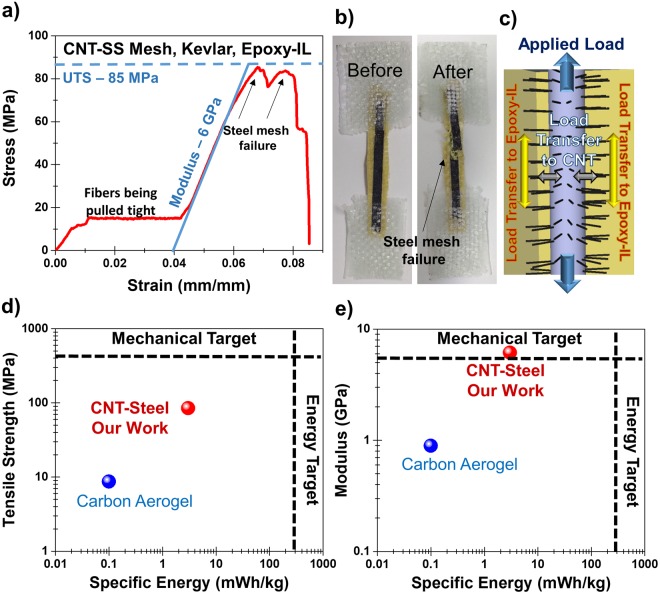
Mechanical performance of the structural supercapacitors and comparison of mechanical vs. electrochemical performance. (**a**) Stress-strain measurement for a structural composite with a Kevlar separator. (**b**) Photographs before and after the tensile testing. (**c**) Schematic highlighting the mechanical failure of the steel mesh. (**d**) Plot of the tensile strength versus the specific energy comparing our devices, literature and the performance targets. (**e**) Plot of the Young’s modulus versus the specific energy comparing our devices, literature, and the performance targets.

Stress-strain measurements for structural composite materials were carried out (Fig. 3a) and demonstrate an initial plateau that corresponds to fibers aligning and pulling taught followed by linear elastic performance of the composite with a calculated modulus of elasticity in this regime of 6.2 GPa. The ultimate tensile strength (UTS) of ~85 MPa, which was the lowest of several tensile tests with a maximum value near 120 MPa, was calculated and the limiting factor in this value was attributed to the steel mesh (Fig. 3b,c) where critical failure occurred. This was confirmed by controlled comparison of the mechanical performance of a CNT-Steel/Kevlar/Epoxy-IL composite to a simpler Kevlar/Epoxy-IL composite (Fig. S12). A key feature of our approach is the presence of CNTs which not only imparts energy storage properties to our composites but also provides effective reinforcement at the interface with the polymer matrix. Numerous previous reports have illuminated the role of CNTs as an effective reinforcing material in polymer composite laminates^51,52^. In particular the research works from John Hart’s’^53^ and Brian Wardle’s^54,55^ research groups have proven the lamellar reinforcement properties of CNTs in polymer composites in some cases providing ~153%^56^ effective reinforcement when compared to the cases without CNTs. For determining interface properties of the as grown CNTs on the stainless steel meshes, we performed lap-joint shear measurements (see Supporting Information, Fig. S13) which indicated increased toughness for the meshes with CNTs when compared to the plain stainless steel meshes. These observations imply that the CNTs in our system perform the dual function of energy storage at the same time providing structural reinforcement to the structural supercapacitor. Further efforts to improve the mechanical properties were carried out including increasing the number of insulating layers between reinforced electrodes and comparing the performance with fiberglass (instead of Kevlar) interlayers (Figs S14 and S15). In this design route, improvements to the mechanical properties occur at the expense of electrochemical properties and increased cell resistance. This becomes increasingly evident when comparing the EIS measurements of the device with 1, 2 and 3 separators (Fig. S16). As the distance between the electrodes increases owing to the increase in the thickness of the separator stack, the equivalent series resistance (ESR) increases. Overall, these mechanical tests imply that routes to grow CNTs on conductive carbon fiber materials instead of stainless steel mesh could lead to a straightforward route to improve mechanical performance, even though such routes are challenging without incurring damage to the carbon fibers^57,58^.

Further comparison of the energy storage and mechanical performance of these devices in the context of carbon aerogels^33^ indicates close to 2 orders of magnitude improvement in energy and power density, 10x and 5x improvement of tensile strength and modulus, respectively (Fig. 3d,e). Targets are also represented on these curves that reflect the minimum performance of a commercial structural composite (~200 MPa) and minimum performance of commercially available electrochemical supercapacitors (~0.5 Wh/kg of total system weight). Whereas our work reaches a critical target for the elastic modulus and provides order(s) of magnitude advancement toward these important performance metrics, greater capacitance and better tensile properties are necessary for the replacement of conventional structural composites with multifunctional energy storage composites using this approach. Moreover, the fiber glass and Kevlar separators used in our work have a mass density of ~83 mg/cm^2^ and ~54 mg/cm^2^ respectively. Adopting separator free approaches as well as using carbon fiber, carbon cloth and other 3D carbon architectures integrated with CNTs are expected to further improve the performance of the total system. These approaches provide a platform for further exploration of alternative pseudocapacitive and faradaic energy storage chemistries which provide a significant increase in energy densities. Our results also indicate that synthetic techniques that could lead to higher surface area CNTs (e.g. SWCNTs) grown on carbon fiber electrodes using the general design approach outlined in this work could ultimately yield performance synergistic with all mechanical and electrochemical targets.

**Figure 4:**
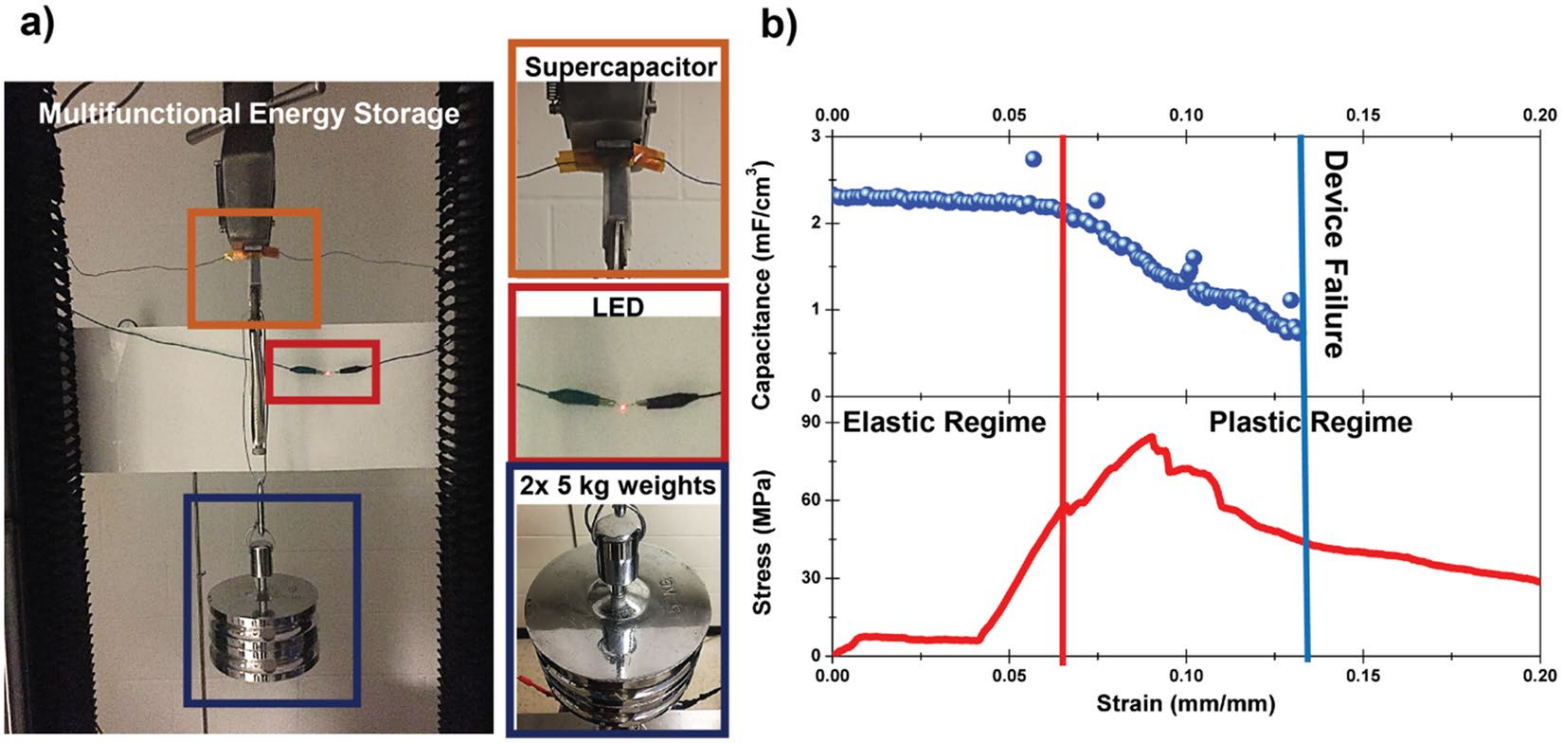
Simultaneous mechanical and electrochemical performance. (**a**) Photograph of a structural supercapacitor material supporting 10 kg of weight in a load cell while simultaneously powering a red LED. (**b**) *In-situ* mechano-electro-chemical measurement showing a concurrent tensile test (bottom) with the measured capacitance from charge discharge curves (top).

To visualize the simultaneous dual functionality of the CNT-steel multifunctional energy composite, an Instron load cell was configured such that two 5 kg weights (10 kg in total) were suspended from a material composed of two structural supercapacitors connected in series, which were discharged to power a red LED (Fig. 4a). Specific description of the testing setup is provided in the Supporting Information Fig. S17. This reflects a consistent observation made in these materials that demonstrates a critical operation condition of a multifunctional energy storage composite, which is to store and release energy while under mechanical stress. To illustrate the potential applicability of this prototype device in load bearing applications, a concrete block (~10 Kg) was suspended attached to the device (see Fig. S18). The device maintained good mechanical integrity providing feasible routes for future work on systems level engineering approaches for integrating such devices into practical load bearing applications. To further mechanistically explore the dual electrochemical-mechanical performance, *in-situ* tests were conducted where energy storage performance and mechanical performance were measured simultaneously (Fig. 4b). Here, within the elastic regime of the material we observe invariant electrochemical and charge storage performance. The supercapacitor shows minimal degradation (~10% capacitance degradation) after 450 charge/discharge cycles during the elastic regime (Fig. S19) with good stability. However, as the device enters the plastic regime, a steady degradation in capacitance is observed until the device is no longer able to charge and complete electrochemical failure occurs. Notably this occurs after the primary mechanical failure of the device near 0.085 strain where the steel mesh begins to deform. The initial degradation of device performance is manifested in increased internal resistance (DCIR) and corresponding greater voltage drop (Fig. S20) associated with the steel electrode failure. To validate the uniformity of this behavior, two similar *in-situ* mechano-electrochemical measurements are presented in Figs S21 and S22 for a composite with plain SS/Kevlar/Epoxy-IL and with a CNT-SS/Fiberglass/Epoxy-IL composite respectively that show similar performance. In each case the electrochemical performance was compromised before the device had fully failed. The results indicate the important concept that electrochemical device failure precedes mechanical device failure, and this occurs at the onset of the plastic regime.

In summary, our work demonstrates a CNT reinforced structural composite supercapacitor that exhibits stable energy storage device behavior under mechanical stress, fulfilling the true function of a structural energy storage composite. This approach involves the reinforcement of an ion-conducting epoxy matrix with CNTs that are grown from tethered sites on a steel mesh electrode and layered with insulating Kevlar or fiberglass materials. Our work utilizes CNTs grown on a conductive structural template as both a mechanically reinforcing interface as well as an electrochemical energy storage architecture for a multifunctional structural energy storage device. Our results indicate elastic modulus above 5 GPa, tensile strength above 85 MPa, and specific energy up to 3 mWh/kg with demonstrated simultaneous energy storage and mechanical performance in the elastic regime. Whereas results of this work bridge critical energy and mechanical targets in comparison to past work, this general technique of utilizing high density CNT growth on conducting surfaces as layered reinforcing interfaces in structural composites reflects a pathway toward composites harnessing performance metrics at the intersection of both commercial composite materials and commercial supercapacitor energy storage devices. With innovation in energy systems continuously driven by greater accessibility and seamless integration of energy into technologies, our results support the extraordinary promise of CNTs as dual reinforcing and energy storage materials in next-generation composite structures.

## Methods

### Growth of CNT on stainless steel mesh

Two types of steel mesh were used in these experiments, a 304 200x200stainless steel mesh (ASTM E2016-06) with a fiber diameter of 53 µm, and a 316 400x400 stainless steel mesh (ASTM E 2016-11) with a fiber diameter of 30.5 µm. CNT growth on these stainless steel meshes was achieved using the same procedure except with small variations in temperature. To grow CNT a three zone furnace was initially heated to the growth temperature. Zones 1 and 2 for preheating the gasses was kept constant at 770 °C and zone 3, the growth zone was varied from 740 °C to 790 °C. The chamber was then purged with 400 SCCM of H_2_ and 100 SCCM of Ar for 15 min. At this point the stainless steel mesh was inserted into the center of zone 3, and annealed under 1000 SCCM of Ar/O_2_ (99%/1%) and 100 SCCM of pure Ar for 45 min. This is accomplished by moving the furnace so that the pre-loaded sample is now in the reaction zone. The Ar/O_2_ was then turned off and the surface was reduced under a flow of 100 SCCM of Ar, and 400 SCCM of H_2_ for 40 min. Finally the growth step was initiated with a gas flow of 100 SCCM of Ethylene, 250 SCCM of Ar/O_2_ (99%/1%), 400 SCCM of H_2_ and 100 SCCM of Ar for 15 min. This growth process was very sensitive to the temperature and we obtained the best growth at a growth temperature of 770 °C for the 304 stainless steel and at 790 °C for the 316 stainless steel^59,60^. Vertically aligned CNT (VACNT) carpets on stainless steel mesh were characterized using scanning electron microscopy (SEM; FEI, Quanta Feg 250) at 5 keV. High resolution transmission electron microscope (HRTEM) samples were prepared by dispersing a section of the CNT carpet in isopropanol with gentle sonication for an hour, and then placing 1 drop of the solution on a 300 mesh Cu lacey carbon grid (from SPI).

### Layup of structural composites

Structural composite supercapacitors were made using a common layup process. Epoxy-ionic liquid (IL) electrolyte was prepared by first dissolving 6 g of lithium tetrafluoroborate (LiBF_4_) in 24 g of 1-butyl 3-methyl imidizolium tetrafluoroborate (BMIBF_4_)^61^. This was followed by mixing supersap CCR epoxy resin with the slow curing hardener in a planetary mixer (Thinky USA). The mixed epoxy and hardener were then combined with the ionic liquid at a ratio of 45–55% epoxy-IL by weight as was determined to be an optimal balance of electrochemical and mechanical performance in previous studies^41^. While the epoxy-IL was still in an uncured state, the epoxy was poured over the CNT-steel mesh and then put under vacuum to infiltrate the epoxy-IL into the CNT. Performance comparison with and without the separate vacuum infiltration step is presented Fig. S23. Following electrolyte infiltration into the CNT-steel mesh, the CNT-Steel mesh was placed against a smooth metal sheet backplate and epoxy-IL was applied to fiberglass (S-Glass, Fiberglasssupply.com) or Kevlar fiber mesh (Fiberglasssupply.com) separators and sandwiched in between the CNT-Steel electrodes. A layer of peel-ply and an absorbing layer were then used to cover the composites and the entire area was sealed and put under vacuum using traditional composite vacuum bagging. The structural composite was either cured overnight at room temperature and then placed on a hot plate at ~50 °C for another 12–24 hours, or placed on a hot plate at 50 °C and left to cure overnight. The latter produced epoxy-IL with the best performance.

### Electrochemical measurements

Electrochemical measurements including cyclic voltammetry (CV) and charge discharge (CD) measurements were performed on an Autolab multichannel analyzer with attached galvanostat/potentiostat modules. CV measurements were performed from −3V to +3 V at scan rates ranging from 20 mV/s to 500 mV/s. In a symmetric two electrode set up, the response between 0 V and 3 V would be similar to that between 0 V and −3V. This test was performed in order to fully assess the stability of the device in this wide voltage range. However, the actual performance of this type of supercapacitive device can be determined using galvanostatic charge discharge tests are performed between 0 V and above (ideally to 2 V in this case where the device performance was stable and minimal unnecessary side reactions were observed). CD measurements were performed with charging currents ranging from 1.5–8 mA/cm^3^. Energy values were calculated by integrating the area under the discharge curves, and average power values were calculated by diving the energy by the discharge time^9^. Values were normalized to the entire mass of the composite including the CNT electrodes, steel mesh current collector, fiberglass or Kevlar separators and the epoxy-IL matrix.

### Mechanical measurements

To perform tensile measurements on the structural supercapacitors the devices were cut in long linear strips about 1 cm x 7 cm in length and then an additional layup step with pure epoxy resin and fiberglass was used to make tabs to enable gripping of the structural composites. Tensile tests were performed with an Instron load cell at a strain rate of 2 mm/min.

*In-situ* mechano-electro-chemical measurements were performed on the same Instron load cell but with a strain rate of 0.5 mm/min and with a portable Autolab potentiostat/galvanostat with alligator clips attached to the respective electrodes. CD measurements were then performed at the same time as the mechanical measurement^41^. A comparison of the mechanical performance at 2 mm/min and at 0.5 mm/min is presented in Fig. S24. Performance is similar but a more abrupt failure occurs in the devices tested at 2 mm/min.

## Electronic supplementary material

Supplementary Information

## References

[1] Gibson, R. F. Principles of composite material mechanics. (CRC press, 2016).

[2] Taraghi I, Fereidoon A, Taheri-Behrooz F (2014). Low-velocity impact response of woven Kevlar/epoxy laminated composites reinforced with multi-walled carbon nanotubes at ambient and low temperatures. Mater. Des.

[3] Salman SD, Leman Z, Sultan MT, Ishak MR, Cardona F (2015). Kenaf/synthetic and Kevlar®/cellulosic fiber-reinforced hybrid composites: A review. BioResources.

[4] Gay, D. Composite materials: design and applications. (CRC press, 2014).

[5] Gojny F, Wichmann M, Köpke U, Fiedler B, Schulte K (2004). Carbon nanotube-reinforced epoxy-composites: enhanced stiffness and fracture toughness at low nanotube content. Compos. Sci. Technol..

[6] Schadler L, Giannaris S, Ajayan P (1998). Load transfer in carbon nanotube epoxy composites. Appl. Phys. Lett..

[7] Chen H (2009). Progress in electrical energy storage system: A critical review. Prog. Nat. Sci..

[8] Dunn B, Kamath H, Tarascon J-M (2011). Electrical energy storage for the grid: a battery of choices. Science.

[9] Cohn AP (2014). Assessing the improved performance of freestanding, flexible graphene and carbon nanotube hybrid foams for lithium ion battery anodes. Nanoscale.

[10] Carter R (2014). Solution assembled single-walled carbon nanotube foams: superior performance in supercapacitors, lithium-ion, and lithium–air batteries. J. Phys. Chem. C.

[11] Salunkhe RR (2015). Large-scale synthesis of coaxial carbon nanotube/Ni (OH) 2 composites for asymmetric supercapacitor application. Nano Energy.

[12] Mohammadi A (2018). Engineering rGO-CNT wrapped Co3S4 nanocomposites for high-performance asymmetric supercapacitors. Chem. Eng. J.

[13] Sengottaiyan C (2017). Cobalt oxide/reduced graphene oxide composite with enhanced electrochemical supercapacitance performance. Bull. Chem. Soc. Jpn..

[14] Sun H (2017). Three-dimensional holey-graphene/niobia composite architectures for ultrahigh-rate energy storage. Science.

[15] Liu J (2014). High-performance flexible asymmetric supercapacitors based on a new graphene foam/carbon nanotube hybrid film. Energy Environ. Sci..

[16] Wang F (2018). Co-doped Ni 3S 2@ CNT arrays anchored on graphite foam with a hierarchical conductive network for high-performance supercapacitors and hydrogen evolution electrodes. J. Mater. Chem. A.

[17] Zhu Y (2012). A seamless three-dimensional carbon nanotube graphene hybrid material. Nat. Commun..

[18] Zhao K (2018). High-yield bottom-up synthesis of 2D metal–organic frameworks and their derived ultrathin carbon nanosheets for energy storage. J. Mater. Chem. A.

[19] He Z (2018). Carbon layer-exfoliated, wettability-enhanced, SO3H-functionalized carbon paper: A superior positive electrode for vanadium redox flow battery. Carbon.

[20] Shi P (2016). Design of amorphous manganese oxide@ multiwalled carbon nanotube fiber for robust solid-state supercapacitor. ACS Nano.

[21] Wu G (2017). High‐Performance Wearable Micro‐Supercapacitors Based on Microfluidic‐Directed Nitrogen‐Doped Graphene Fiber Electrodes. Adv. Funct. Mater..

[22] Wang W (2015). A novel exfoliation strategy to significantly boost the energy storage capability of commercial carbon cloth. Adv. Mater..

[23] Thomas, J. P., Keennon, M. T., DuPasquier, A., Qidwai, M. A. & Matic, P. Multifunctional Structure-Battery Materials for Enhanced Performance in Small Unmanned Air Vehicles. *Proc*. *of the 2003 International Mechanical Engineering Congress and Exposition*, Washington, DC, USA. Paper no. IMECE2003-41512, pp. 289–292, 10.1115/IMECE2003-41512 (2003).

[24] Qidwai, M. A., Thomas, J. & Matic, P. Structure-battery multifunctional composite design. *Proc*. *SPIE 9th Annual International Symposium on Smart Structures and Materials*, San Diego, California, USA. Volume 4698. pp. 180–191, 10.1117/12.475063 (2002).

[25] Thomas J, Qidwai S, Pogue W, Pham G (2013). Multifunctional structure-battery composites for marine systems. J. Compos. Mater..

[26] Liu P, Sherman E, Jacobsen A (2009). Design and fabrication of multifunctional structural batteries. J. Power Sources.

[27] Wetzel, E. D., O’Brien, D. J., Snyder, J. F., Carter, R. H. & South, J. T. Multifunctional structural power and energy composites for US army applications. *Multifunctional Structures*/*Integration of Sensors and Antennas Conference*, Neuilly-sur-Seine, France. Paper number RTO-MP-AVT-141, Paper 2, pp. 2-1–2-14 (2006).

[28] Shirshova N (2014). Multifunctional structural energy storage composite supercapacitors. Faraday Discuss..

[29] Benson J (2013). Multifunctional CNT‐Polymer Composites for Ultra‐Tough Structural Supercapacitors and Desalination Devices. Adv. Mater..

[30] Qian H (2013). Activation of structural carbon fibres for potential applications in multifunctional structural supercapacitors. J. Colloid Interface Sci..

[31] Javaid A (2014). Multifunctional structural supercapacitors for electrical energy storage applications. J. Compos. Mater..

[32] Shirshova N (2013). Structural composite supercapacitors. Compos. Part A Appl. Sci. Manuf..

[33] Qian H, Kucernak AR, Greenhalgh ES, Bismarck A, Shaffer MS (2013). Multifunctional structural supercapacitor composites based on carbon aerogel modified high performance carbon fiber fabric. ACS Appl. Mater. Interfaces.

[34] Deka, B. K., Hazarika, A., Kim, J., Park, Y. B. & Park, H. W. Recent development and challenges of multifunctional structural supercapacitors for automotive industries. *International Journal of Energy Research* (2017).

[35] Deka BK, Hazarika A, Kim J, Park Y-B, Park HW (2016). Multifunctional CuO nanowire embodied structural supercapacitor based on woven carbon fiber/ionic liquid–polyester resin. Composites Part A: Applied Science and Manufacturing.

[36] Hudak NS, Schlichting AD, Eisenbeiser K (2017). Structural Supercapacitors with Enhanced Performance Using Carbon Nanotubes and Polyaniline. Journal of The Electrochemical Society.

[37] Wang Y, Qiao X, Zhang C, Zhou X (2016). Development of All-Solid-State Structural Supercapacitor Using an Epoxy Based Adhesive Polymer Electrolyte. ECS Transactions.

[38] Yang XF (2016). 1-D oriented cross-linking hierarchical porous carbon fibers as a sulfur immobilizer for high performance lithium-sulfur batteries. Journal of Materials Chemistry A.

[39] Senokos, E. *et al*. Large‐Area, All‐Solid, and Flexible Electric Double Layer Capacitors Based on CNT Fiber Electrodes and Polymer Electrolytes. *Adv*. *Mater*.*Technol*. **2** (2017).

[40] Wu G (2017). High-performance supercapacitors based on electrochemical-induced vertical-aligned carbon nanotubes and polyaniline nanocomposite electrodes. Sci Rep..

[41] Westover AS (2015). Multifunctional high strength and high energy epoxy composite structural supercapacitors with wet-dry operational stability. J. Mater. Chem. A.

[42] Westover AS (2014). Stretching ion conducting polymer electrolytes: *in-situ* correlation of mechanical, ionic transport, and optical properties. J. Electrochem. Soc..

[43] Wang G, Zhang L, Zhang J (2012). A review of electrode materials for electrochemical supercapacitors. Chem. Soc. Rev..

[44] Asp LE, Greenhalgh ES (2014). Structural power composites. Compos. Sci. Technol.

[45] Hashempour M, Vicenzo A, Zhao F, Bestetti M (2013). Direct growth of MWCNTs on 316 stainless steel by chemical vapor deposition: Effect of surface nano-features on CNT growth and structure. Carbon.

[46] Camilli L (2011). The synthesis and characterization of carbon nanotubes grown by chemical vapor deposition using a stainless steel catalyst. Carbon.

[47] Masarapu C, Wei B (2007). Direct growth of aligned multiwalled carbon nanotubes on treated stainless steel substrates. Langmuir.

[48] Wu Y, Qiao P, Chong T, Shen Z (2002). Carbon nanowalls grown by microwave plasma enhanced chemical vapor deposition. Adv. Mater..

[49] Westover AS (2014). A multifunctional load-bearing solid-state supercapacitor. Nano Lett..

[50] Senokos E (2018). Energy storage in structural composites by introducing CNT fiber/polymer electrolyte interleaves. Sci Rep.

[51] Soliman EM, Kandil UF, Reda Taha MM (2014). Investigation of FRP lap splice using epoxy containing carbon nanotubes. J. Compos. Constr..

[52] Garcia EJ, Wardle BL, Hart AJ, Yamamoto N (2008). Fabrication and multifunctional properties of a hybrid laminate with aligned carbon nanotubes grown *in situ*. Compos. Sci. Technol..

[53] Garcia EJ, Wardle BL, Hart AJ (2008). Joining prepreg composite interfaces with aligned carbon nanotubes. Compos. Pt. A. Appl. Sci. Manuf.

[54] Blanco J, García EJ, Guzmán de Villoria R, Wardle BL (2009). Limiting mechanisms of mode I interlaminar toughening of composites reinforced with aligned carbon nanotubes. J. Compos. Mater..

[55] Wicks SS, de Villoria RG, Wardle BL (2010). Interlaminar and intralaminar reinforcement of composite laminates with aligned carbon nanotubes. Compos. Sci. Technol.

[56] Garcia, E. J., Hart, A. J., Wardle, B. L., Slocum, A. H. & Shim, D.-J. Aligned carbon nanotube reinforcement of graphite/epoxy ply interfaces. *16th International Conference on Composite Materials*, *Kioto*, *Japan* (2007).

[57] Pint CL, Alvarez NT, Hauge RH (2009). Odako growth of dense arrays of single-walled carbon nanotubes attached to carbon surfaces. Nano Research.

[58] Davis BF (2016). Electrically Conductive Hierarchical Carbon Nanotube Networks with Tunable Mechanical Response. ACS Applied Materials & Interfaces.

[59] Teblum E (2014). Millimeter-Tall Carpets of Vertically Aligned Crystalline Carbon Nanotubes Synthesized on Copper Substrates for Electrical Applications. J. Phys. Chem. C.

[60] Teblum E (2016). Differential preheating of hydrocarbon decomposition and water vapor formation shows that single ring aromatic hydrocarbons enhance vertically aligned carbon nanotubes growth. Carbon.

[61] Shirshova N (2013). Structural supercapacitor electrolytes based on bicontinuous ionic liquid–epoxy resin systems. J. Mater. Chem. A.

