# Supplementary material for: Carbon Nanotube Reinforced Structural Composite Supercapacitor
Source: Sci Rep. 2018 Dec 5;8:17662. doi: 10.1038/s41598-018-34963-x (PMC6281659; doi:10.1038/s41598-018-34963-x)
Supplement: Supplementary file 1 — Supplementary Information [file 41598_2018_34963_MOESM1_ESM.docx]

**Supplementary Information: Carbon Nanotube Reinforced Structural Composite Supercapacitor**

*Nitin Muralidharan,^1,2,#^ Eti Teblum,^3,#^ Andrew Westover,^1,2,#^ Deanna Schauben,^1^ Anat Yitzhak ,^3^ Merav Muallem^3^ Gilbert D. Nessim,^3^ Cary L. Pint^1,2,^**

^1^Department of Mechanical Engineering, Vanderbilt University, Nashville TN 37235, USA ^2^Interdisciplinary Materials Science Program, Vanderbilt University, Nashville, TN 37235, USA ^3^Department of Chemistry, Bar Ilan Institute for Nanotechnology and Advanced Materials (BINA), Bar Ilan University, 52900 Ramat Gan, Israel


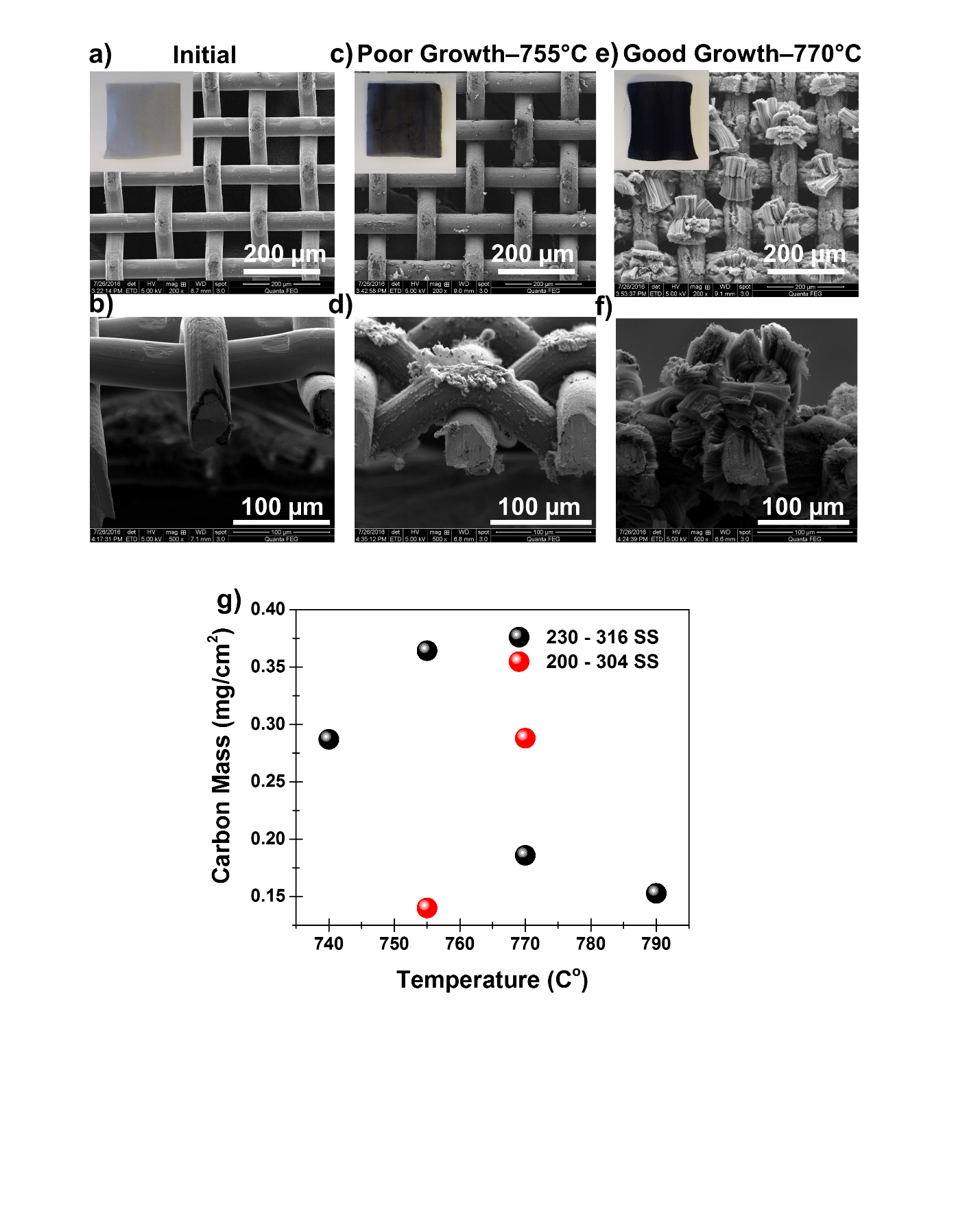


**Figure S1:** **CNT growth for different temperatures** a) Bird’s eye view SEM image of pristine stainless steel mesh. b) Cross-sectional SEM image of pristine stainless steel mesh. c) Bird’s eye view SEM image of CNT- stainless steel mesh grown at 755°C. d) Cross-sectional SEM image of CNT- stainless steel mesh grown at 755°C. e) Bird’s eye view SEM image of CNT- stainless steel mesh grown at 770°C. f) Cross-sectional SEM image of CNT- stainless steel mesh grown at 770°C. Generally, CNT heights are measured between 15 – 80 µm.


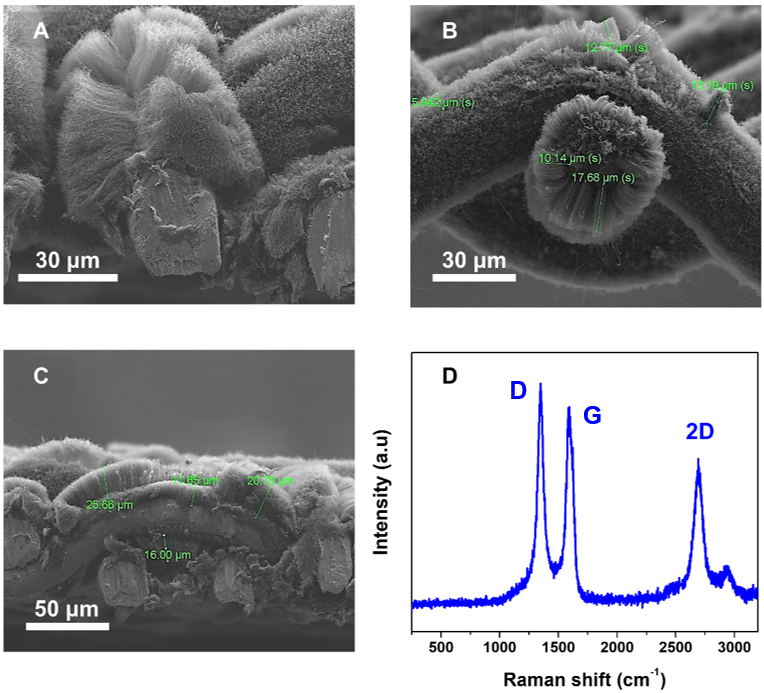


**Figure S2: Microstructural and spectroscopic characterization of the CNTs grown on stainless steel meshes.** (a-c) SEM micrographs of the CNTs, b) Raman spectra of the CNTs grown on stainless steel meshes.


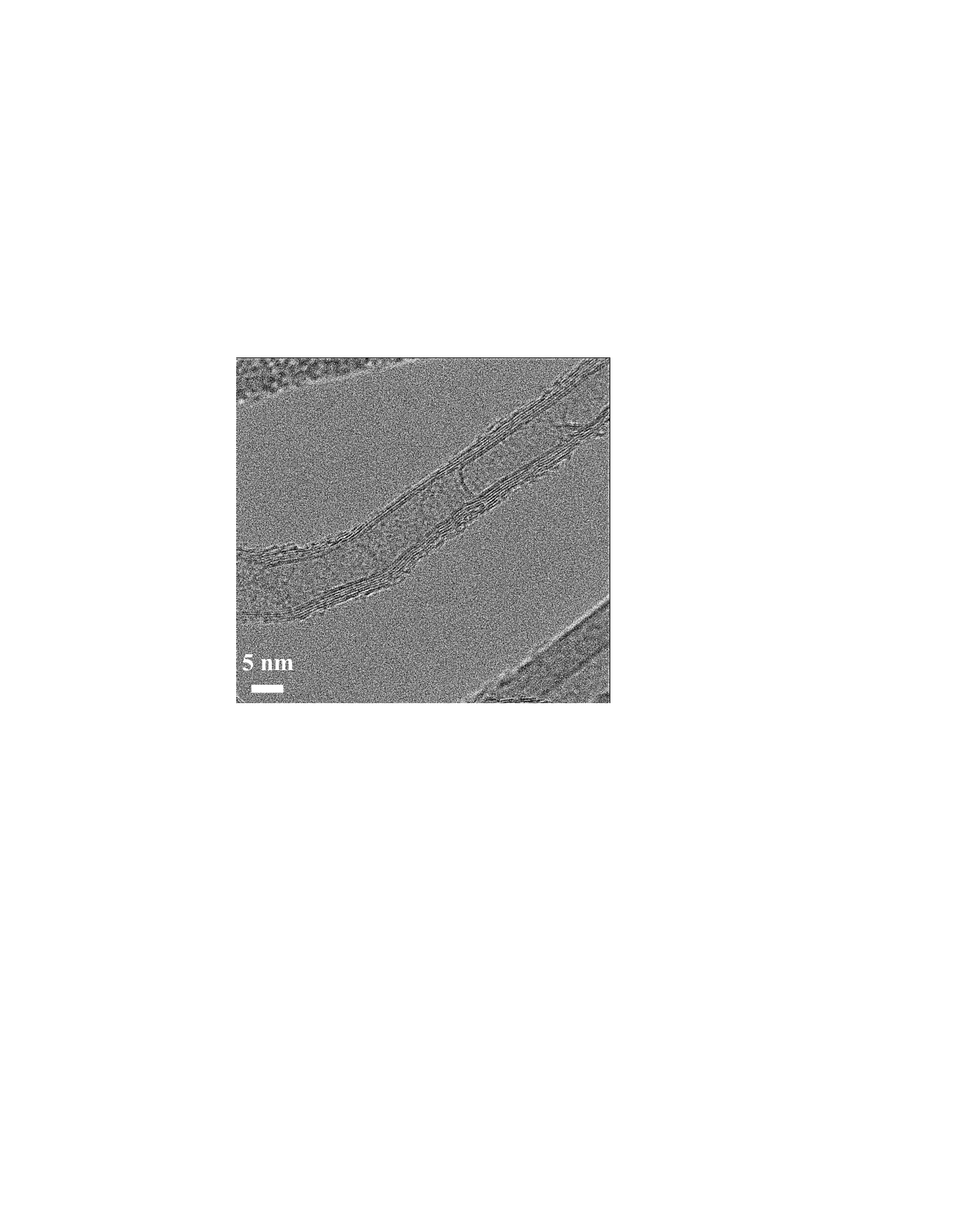


**Figure S3: TEM image of CNT grown on stainless steel mesh**

**Mechanism Controlling CNT Synthesis on SS Meshes**

Synthesis of VACNTs on stainless steel mesh includes oxidation and reduction processes of the mesh following by CNTs growth, as explained in detail by prior studies by Karwa et al..^1^ Pretreatment of the metallic surface of the mesh prior to CNTs growth step is crucial to achieving successful growth using this technique. Oxidation of the stainless steel surface by oxygen flow (ppm) at higher temperatures (770 and 790 °C) initiates the breakup of the metallic surface (Fe, Ni) into distinct granular structures that serve to function as catalyst dots or particles. Activation of Fe catalyst dots by a reduction process (hydrogen flow) before CNTs growth step is needed for enhancing the catalytic activity by turning oxidized Fe to its zero-valent (metallic) state (Teblum et. al.).^2^

**
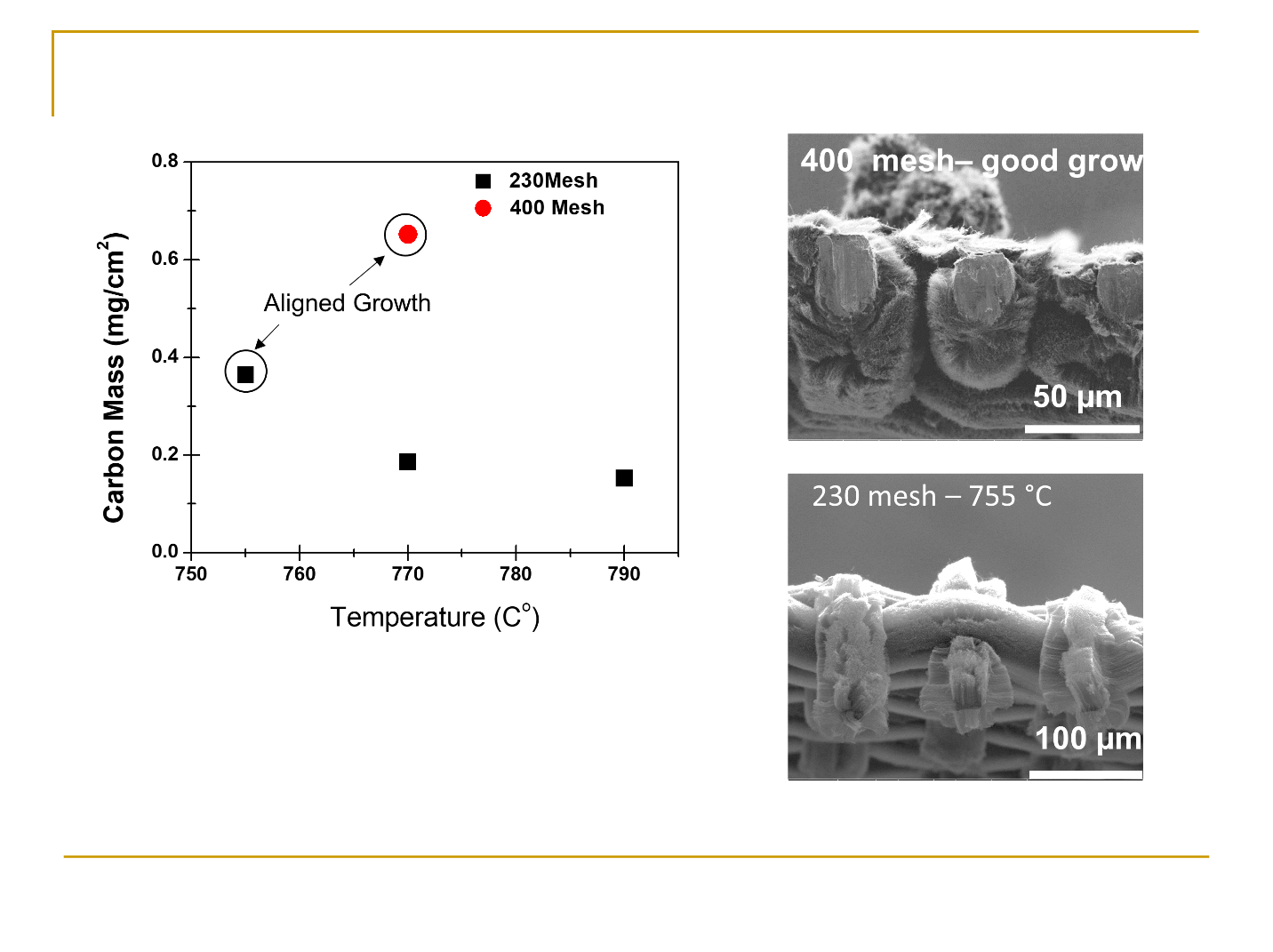
**

**Figure S4:** **Comparison of CNTs grown on 230 mesh and 400 mesh stainless steel samples.** Mass of the carbon nanotubes grown and information regarding the nature of growth is provided.


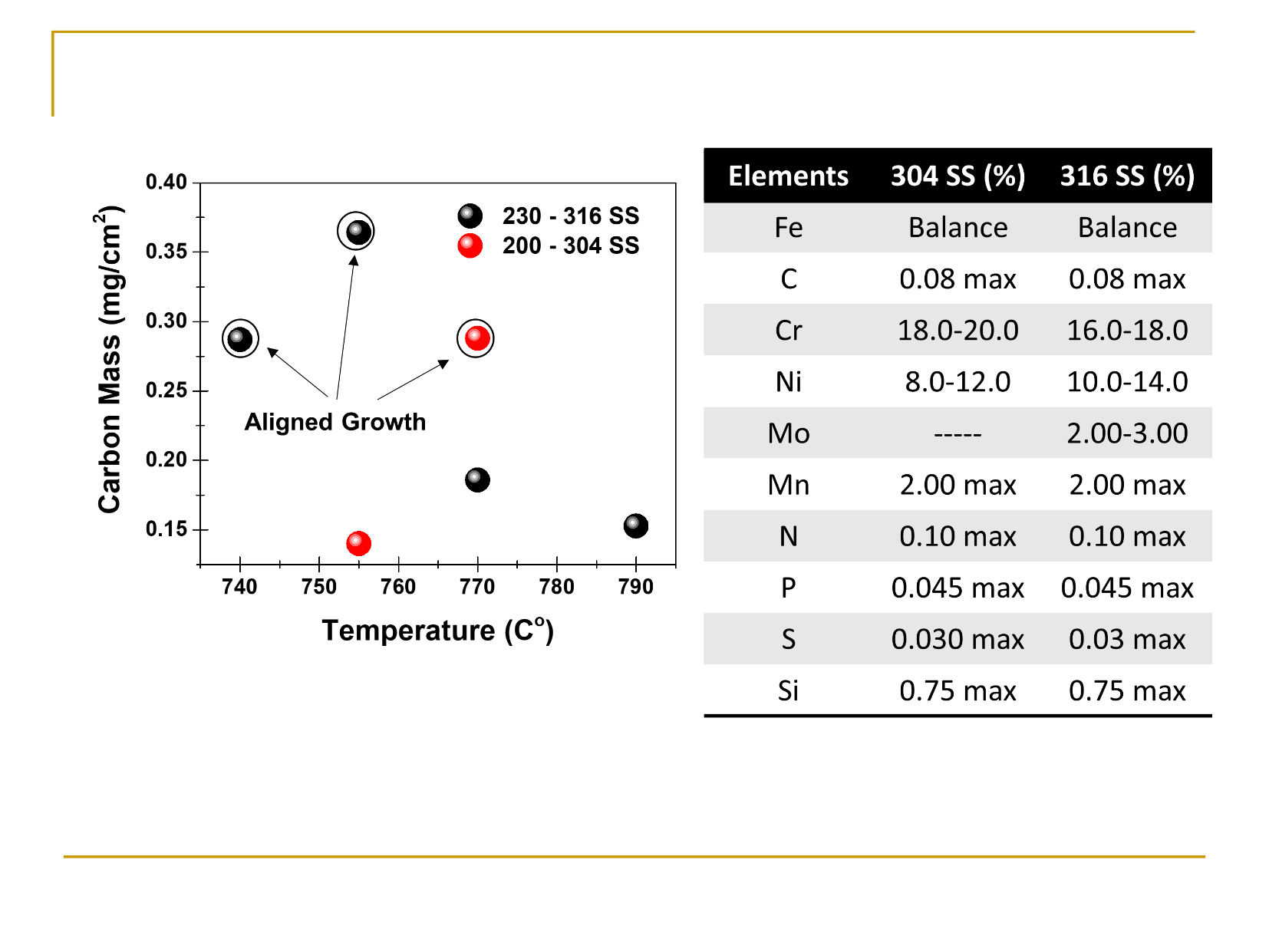


**Figure S5:** **Comparison of CNTs grown on 316 and 304 stainless steel samples.** Mass of the carbon nanotubes grown and information regarding the nature of growth is provided.

| **Elements** | **304 SS (%)** | **316 SS (%)** |
| --- | --- | --- |
| Fe | Balance | Balance |
| C | 0.08 max | 0.08 max |
| Cr | 18.0-20.0 | 16.0-18.0 |
| Ni | 8.0-12.0 | 10.0-14.0 |
| Mo | ----- | 2.00-3.00 |
| Mn | 2.00 max | 2.00 max |
| N | 0.10 max | 0.10 max |
| P | 0.045 max | 0.045 max |
| S | 0.030 max | 0.03 max |
| Si | 0.75 max | 0.75 max |

**Table S6:** **Comparison of elemental composition of the 304 and 316 steel meshes.**


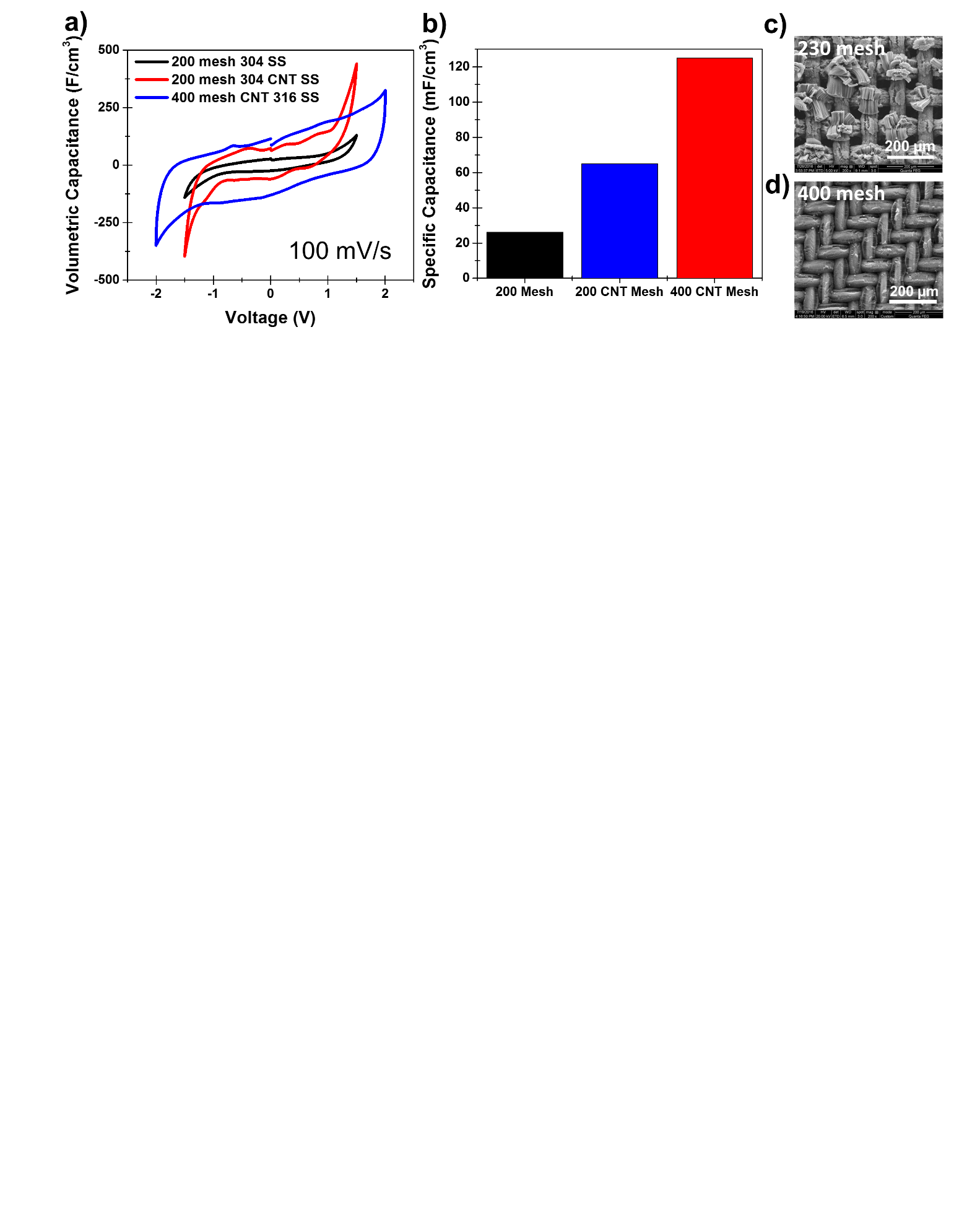


**Figure S7:** **Comparison of CNT grown on 304 SS 230 mesh and 316 SS 400 mesh in IL.**  a) CV curves for a plain 304 SS mesh (black), CNT grown on 304 SS mesh (red), and CNT grown on a 316 SS mesh (blue) in pure ionic liquid and at a scan rate of 100 mV/s. b) A comparison of the capacitance of the above samples. c) SEM image of the CNT grown on the 304 SS 200 mesh. d) SEM image of CNT grown on the 316 SS 400 mesh.


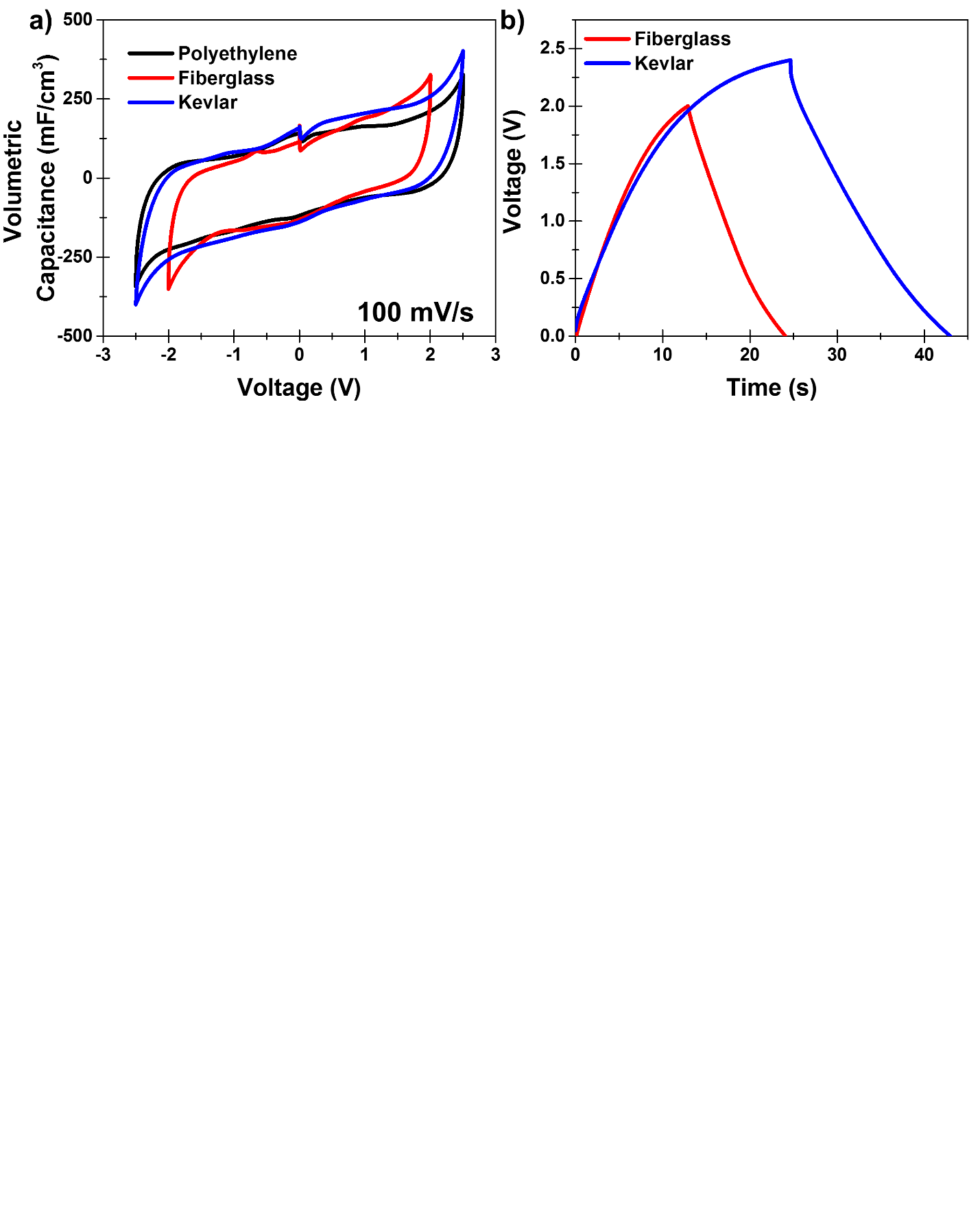


**Figure S8:** **Comparison of fiberglass and Kevlar separators in IL.**  a) CV curves of 316 SS 400 mesh with a traditional polyethylene, fiberglass and Kevlar separator. b) CD curves with fiberglass and Kevlar separators.


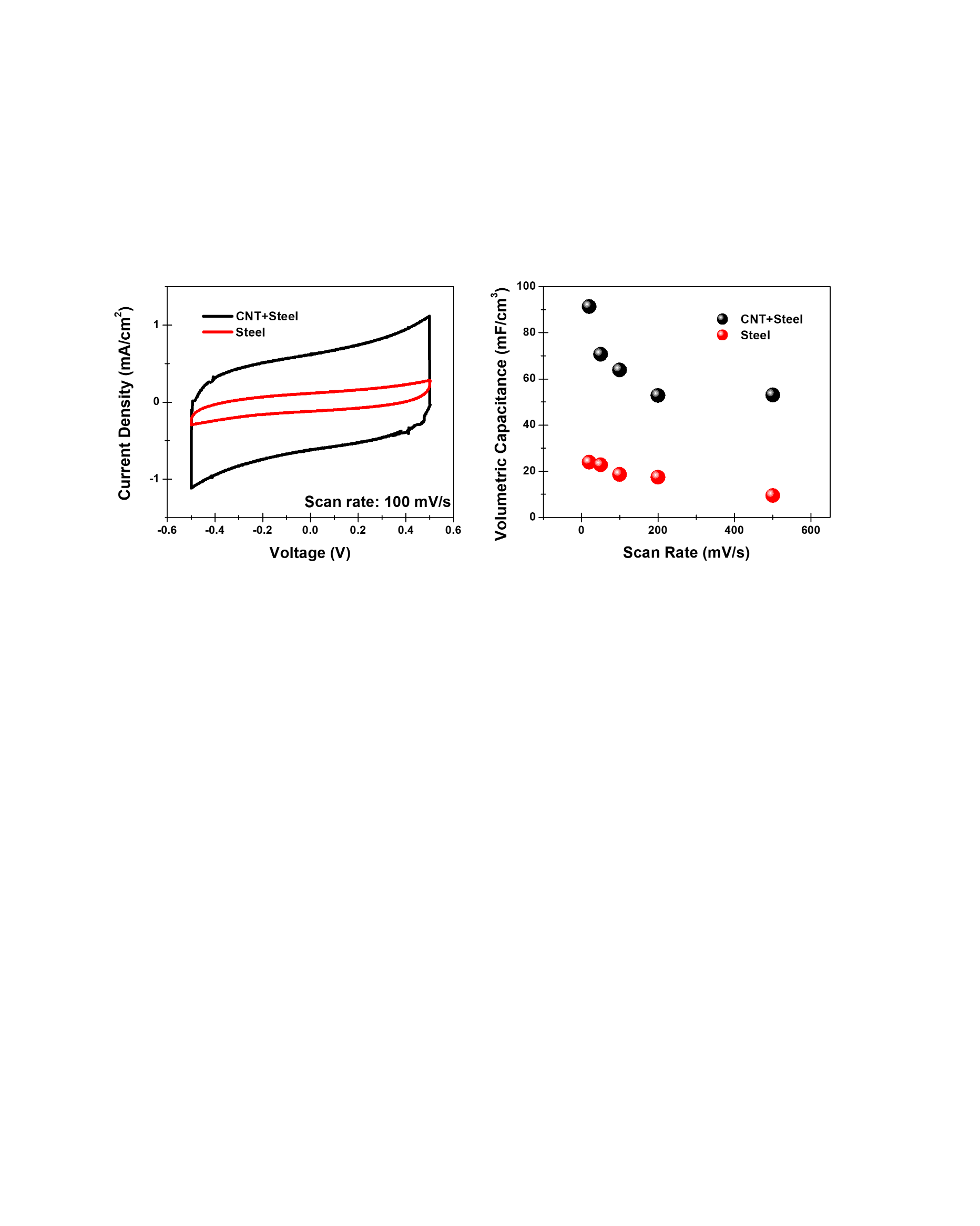


**Figure S9:** **Comparison of electrochemical performance of stainless steel meshes with and without CNTs.**  a) CV curves at 100 mV/s. b) Volumetric capacitance at various scan rates.


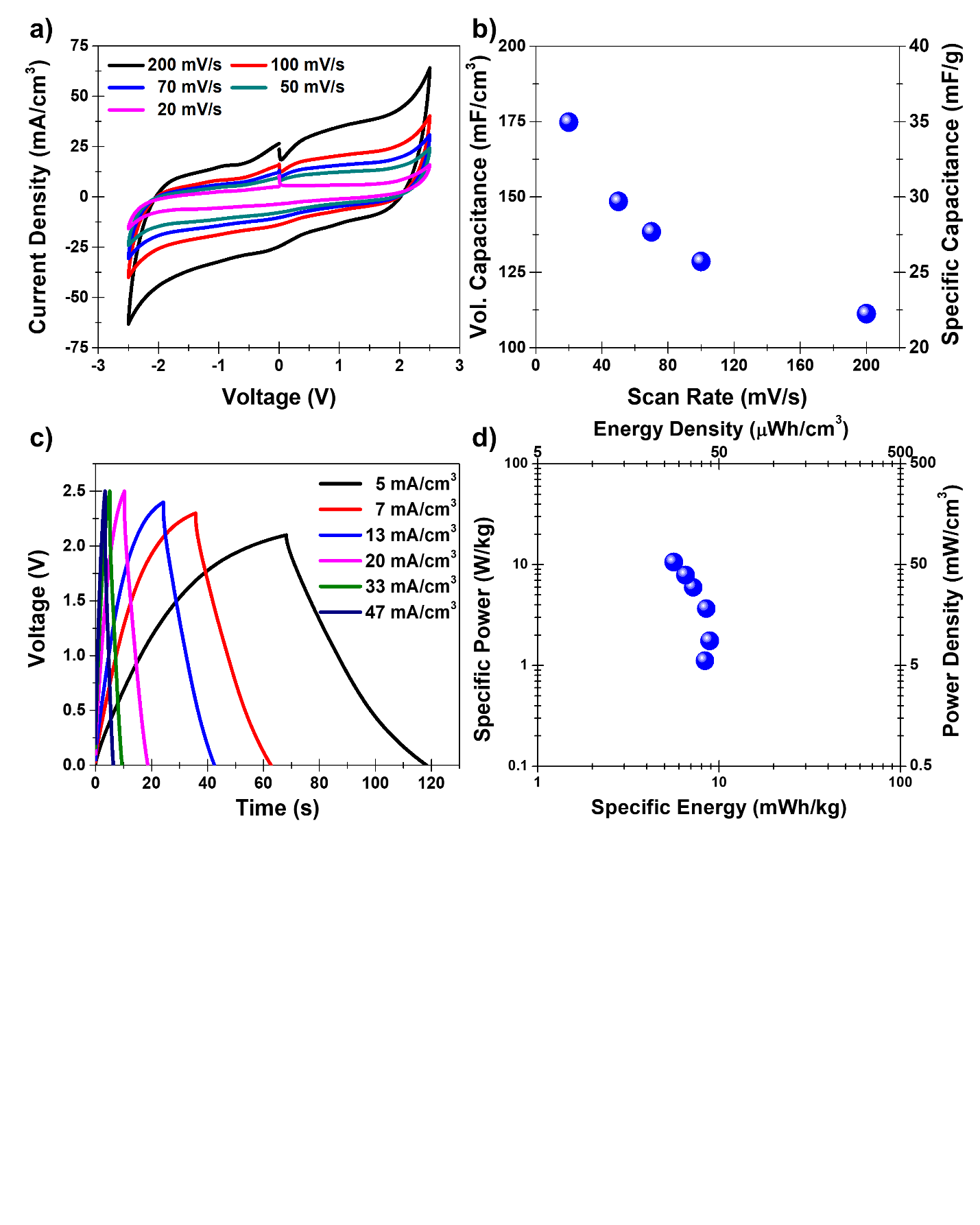


**Figure S10:** **Full electrochemical characterization of CNT on 316 SS 400 mesh with Kevlar separator and pure IL electrolyte.**  a) CV curves of 316 SS 400 mesh with a Kevlar separator and a pure IL electrolyte for scan rates ranging from 20-200 mV/s. b) Capacitance as measured from CV curves in a). c) CD for the same device in a) for charging currents from 5 mA/cm^3^ to 50 mA/cm^3^. d) Ragone plot calculated from the CD curves in c).

| **Composite** | **Gravimetric Energy Density (mWh/kg)** | **Volumetric Energy Density**  **(µWh/cm^3^)** | **Gravimetric Power Density (W/kg)** | **Volumetric Power Density**  **(mW/cm^3^)** |
| --- | --- | --- | --- | --- |
| Our Work  (Solid state) | 3 | 10 | 1 | 70 |
| Our Work  (Liquid state) | 10 | 30 | 10 | 700 |
| Carbon Aerogel (Solid)^3^ | ~0.9 | ~0.18 | ~0.003 | ~0.007 |
| Carbon Aerogel  (Liquid)^3^ | ~1 | ~2.7 | ~0.3 | ~0.6 |
| CF-Epoxy^4^ | 98.9 | - | 3.84 | - |
| CF-Polyester^5^ | 42.15 | - | 4.14 | - |
| CF-PAN^6^ | 10 | - | 71 | - |
| CNT Fiber^7^ | 37.5 | - | 30 | - |

**Table S11: Summary of energy and power densities of the various state of the art structural supercapacitors in literature compared to our device.**


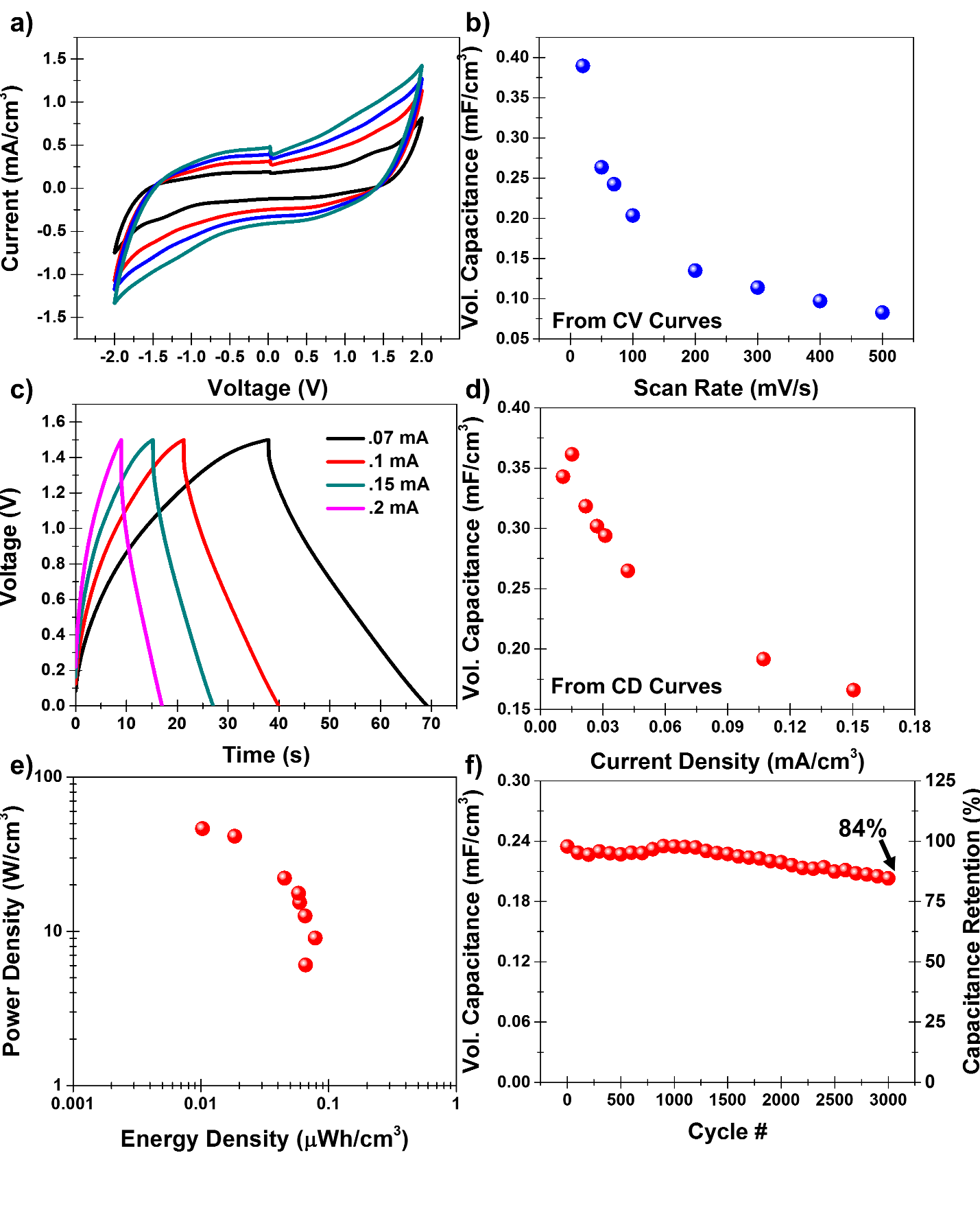


**Figure S12:** **Full electrochemical characterization of plain SS mesh a fiberglass separator and an epoxy-IL electrolyte.** a) CV curves of 304 SS 200 mesh with a fiberglass separator and a 45-55 epoxy-IL structural electrolyte for scan rates ranging from 20-500 mV/s. b) Capacitance as measured from CV curves in a). c) CD for the same device in a). d) Capacitance as measured from CD curves. e) Ragone plot calculated from the CD curves in c). f) Cycling data for the device over 3000 cycles.


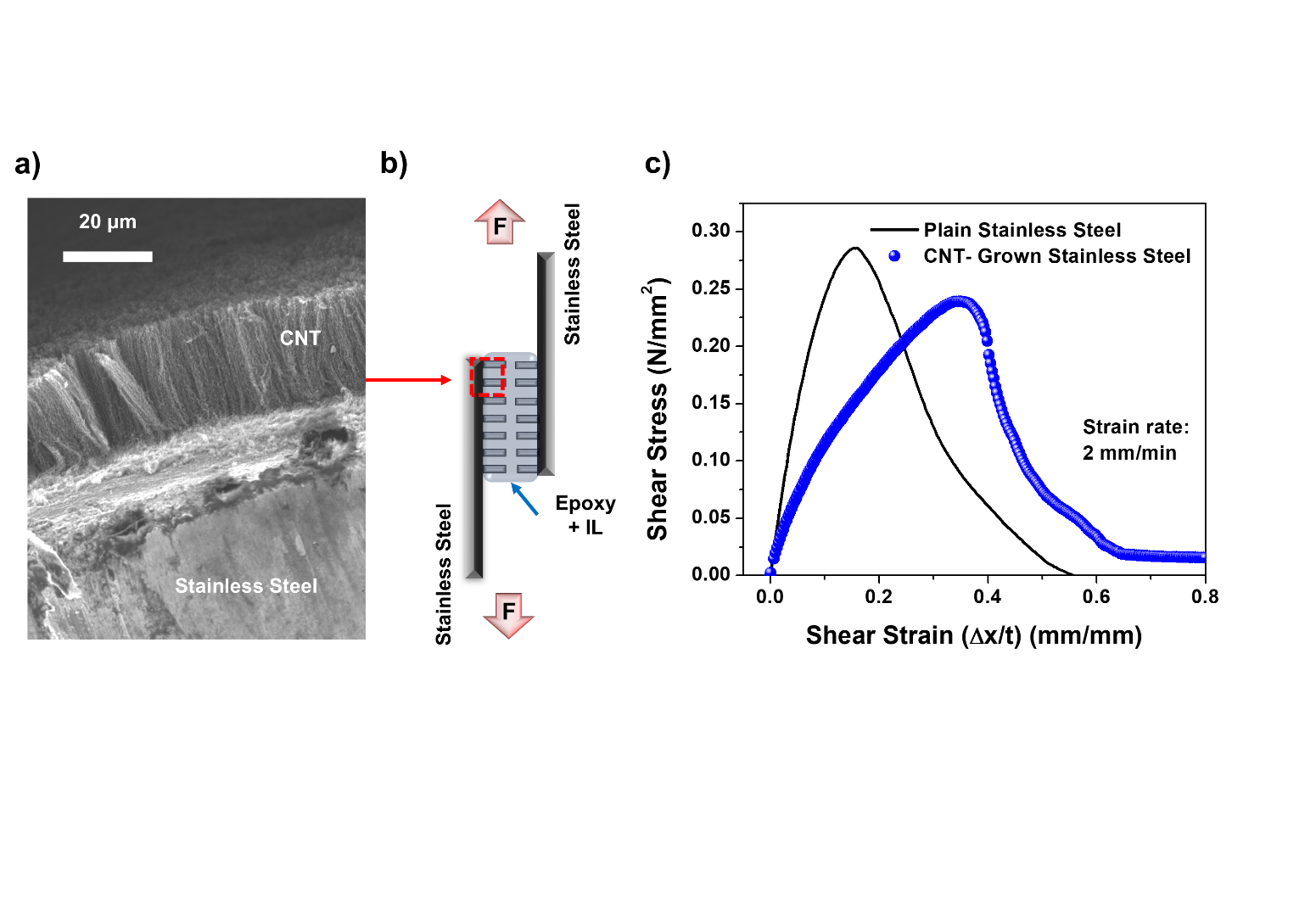


**Figure S13: Lap-joint shear test to determine interface reinforcement.** a) SEM cross-sectional image of CNTs grown on stainless steel sheets for analyzing the reinforcement at the interface. b) Schematic representation of the methodology of the lap-joint shear test. c) The shear stress against shear strain response of the plain stainless steel sample when compared to the CNT-grown stainless steel sample showing increased shear toughness (area encompassed by the curve) for the CNT grown samples (strain rate: 2 mm/min).

For analyzing interface reinforcement effects, CNTs were grown on stainless steel sheets (Type – 316) as observed from the cross-sectional SEM image shown in Figure S9. a. Following this, lap-joint shear tests (Figure S11. b) were performed on the CNT-grown stainless steel samples as well as plain stainless steel samples as a control. Epoxy-Ionic liquid (55 wt%:45 wt%) was used as the polymer matrix to facilitate better detection of interface properties. The shear measurements were performed on a Instron 5944 mechanical testing system at a standard strain rate of 2 mm/min. The test parameters and polymer/IL ratios were chosen such that the reinforcing effect of CNTs at the interface of the polymer matrix can be effectively isolated. The shear toughness of the samples was calculated from the area encompassed by the stress-strain curve (Figure S9. c) before failure (minimum load detection). Comparing the best samples, we observe that the plain stainless steel sample had a shear toughness value of 0.0073 J/mm^3^ when compared to the CNT grown stainless steel which had a value of 0.0082 J/mm^3^. These tests indicated a ~12% increase in toughness for the samples which had the CNTs reinforcing the interface.


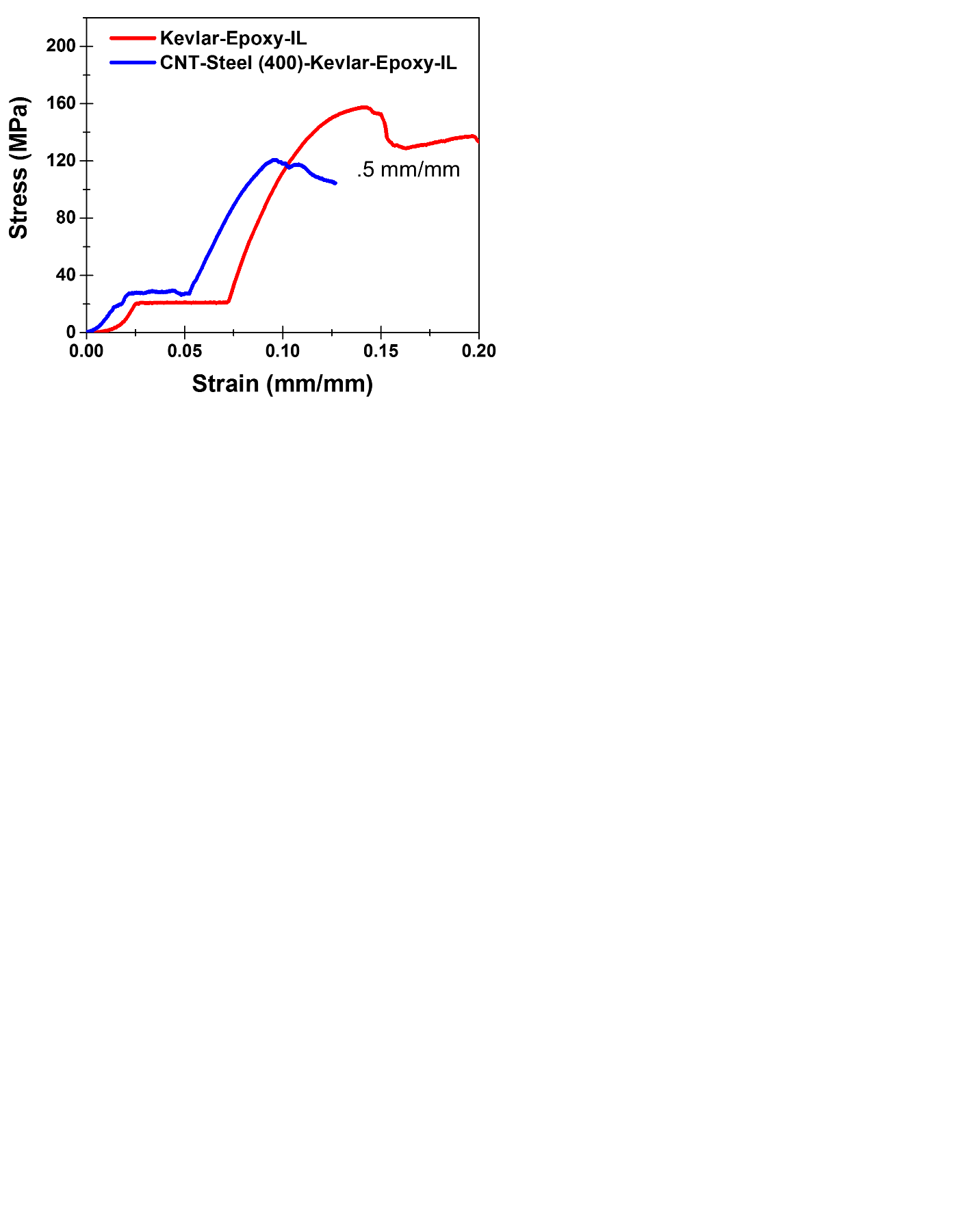


**Figure S14:** **Stress-strain curves of structural composites with and without CNT-Steel.**  Stress-strain curves of a Kevlar-Epoxy-IL composite (red) and a CNT grown on 400 mesh SS/Kevlar/Epoxy-IL composite (blue) at a scan rate of .5 mm/min.

**Figure S15:** **Stress strain curve of different separators.** Stress strain curve of stainless steel (200 mesh) /Kevlar (black) and fiberglass (red)/Epoxy-IL structural supercapacitors.


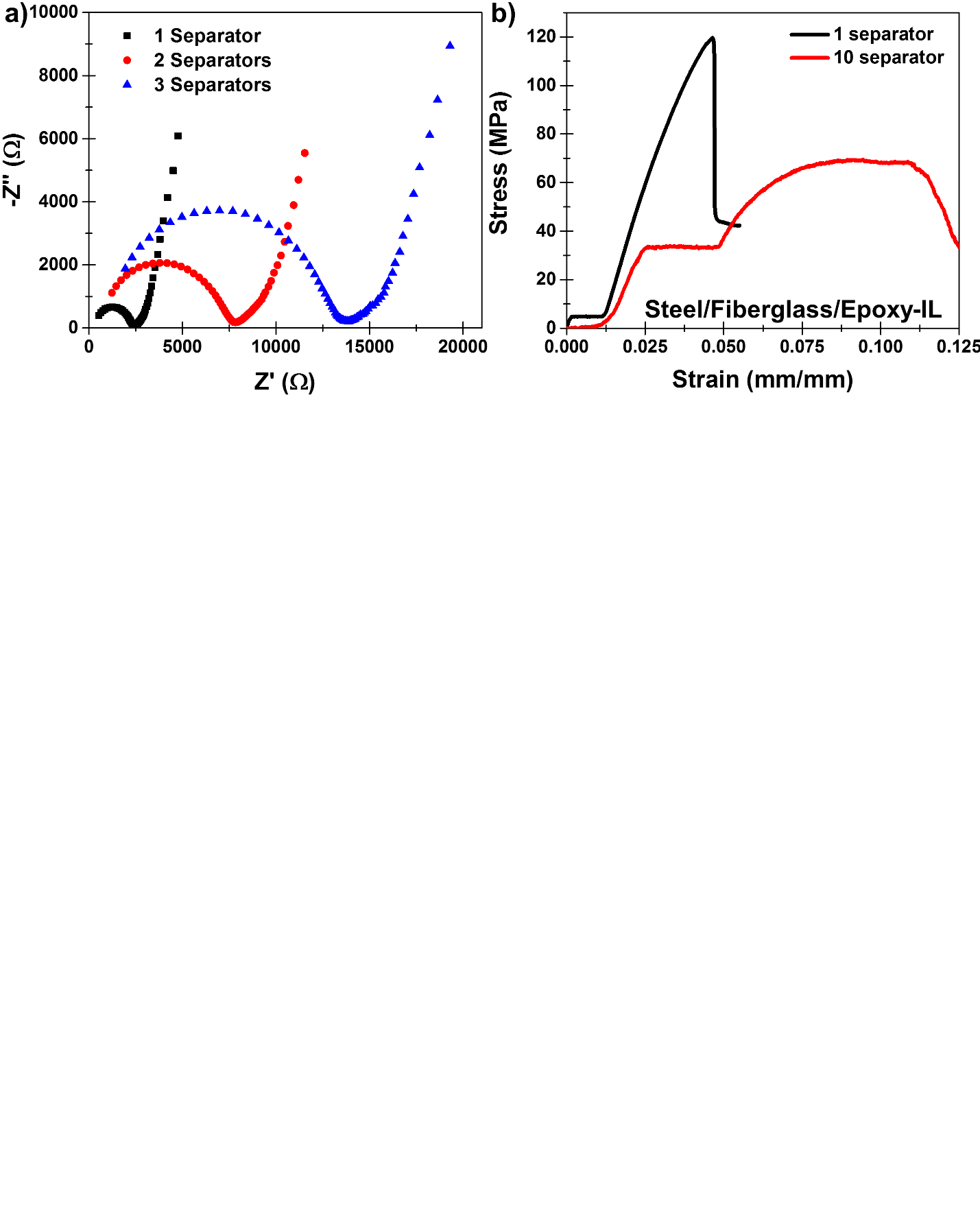


**Figure S16:** **Performance with different numbers of fiberglass separators.** a) EIS performance of CNT grown on 304 SS 200 mesh with 1, 2 and 3 fiberglass separators showing increasing resistance for more separators. b) Stress-strain performance of 304 SS mesh composites with 1 and 10 separators.


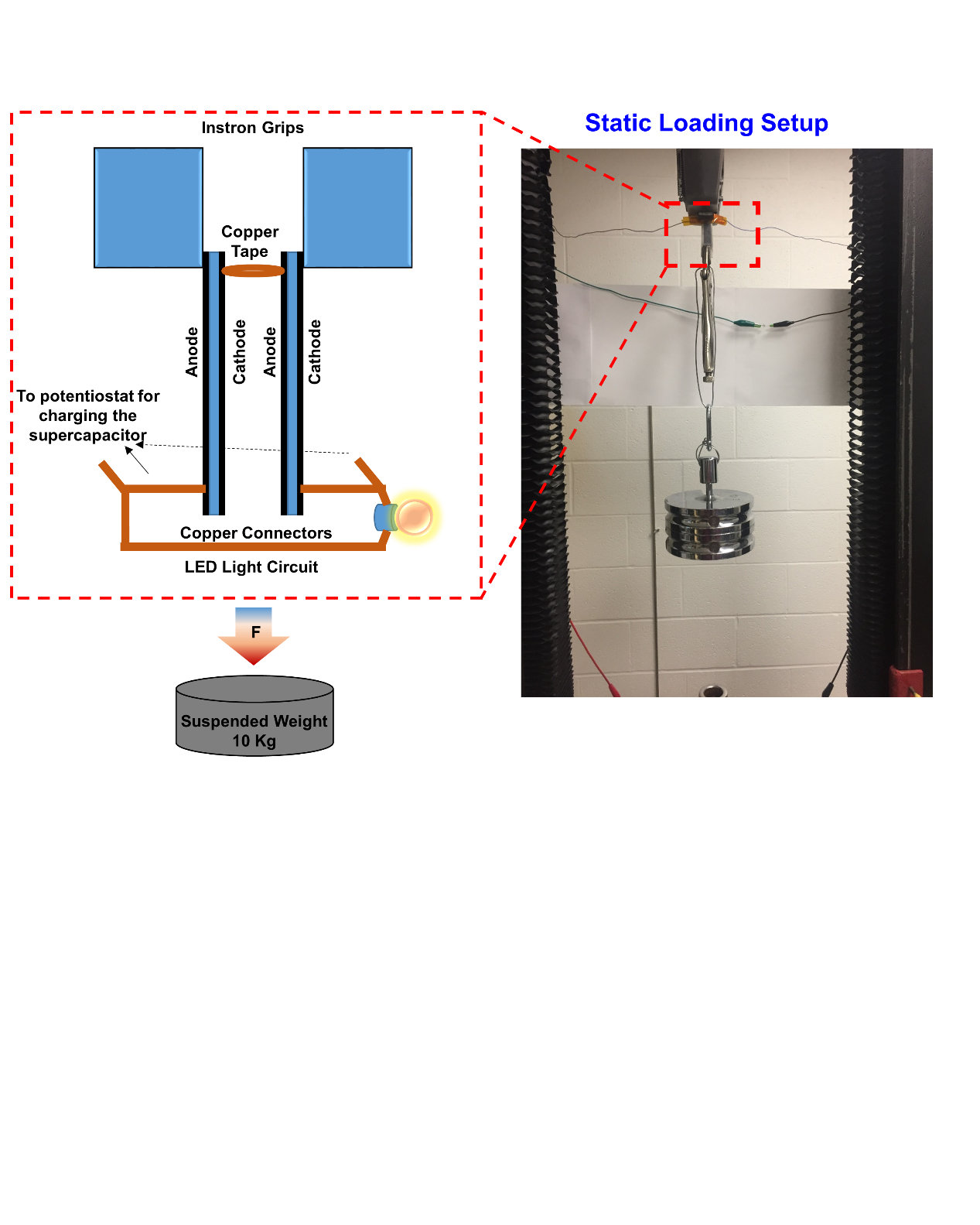


**Figure S17: Static loading using a concrete block testing setup.** Photograph of a structural supercapacitor material supporting ~10 kg of weight concrete block.

Two supercapacitive composites were connected in series using double sided copper tapes and copper connectors as shown in the figure. The device was charged using a potentiostat and was discharged to power an LED. During the discharging process, the power supply from the potentiostat was turned off.


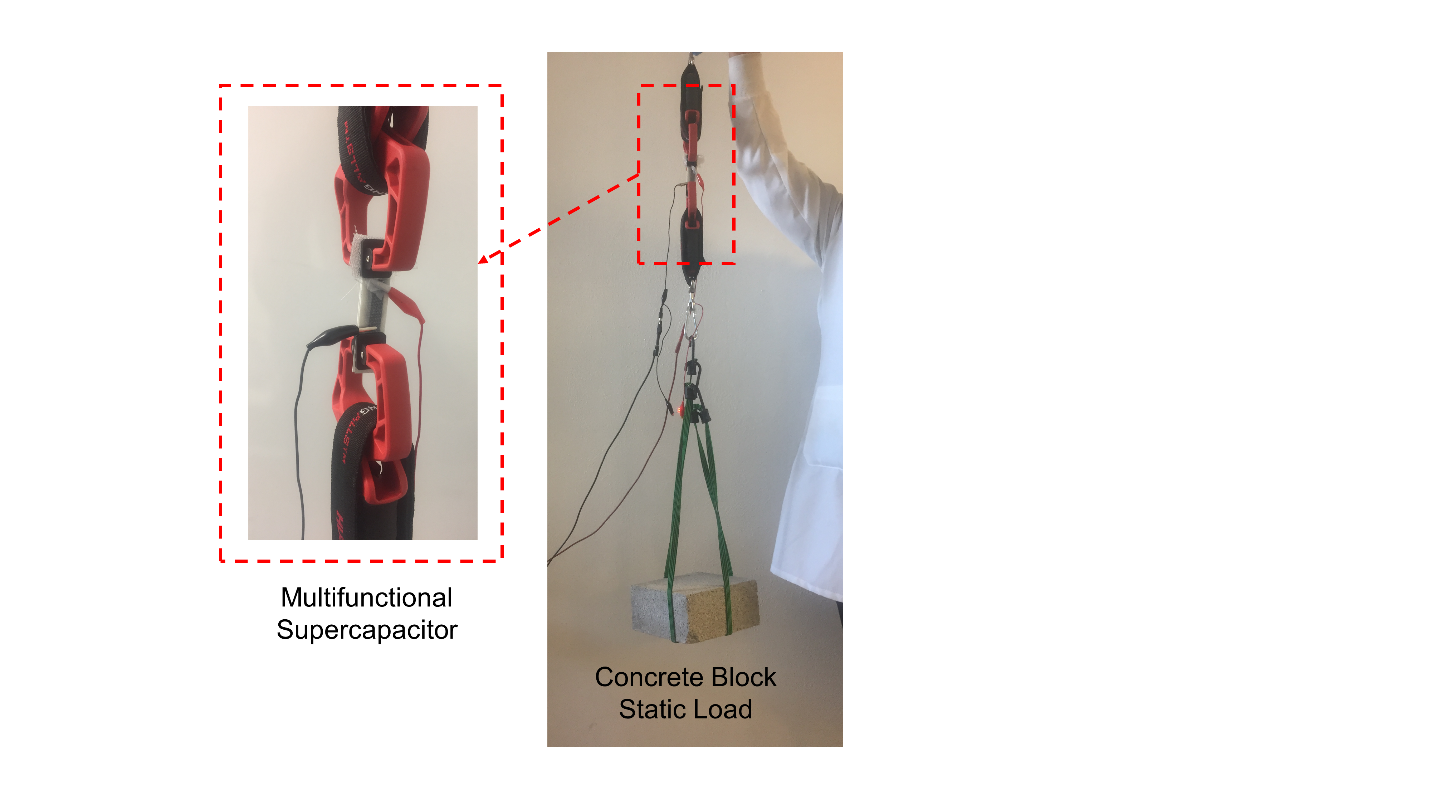


**Figure S18: Static loading using a concrete block.** Photograph of a structural supercapacitor material supporting ~10 kg of weight concrete block.


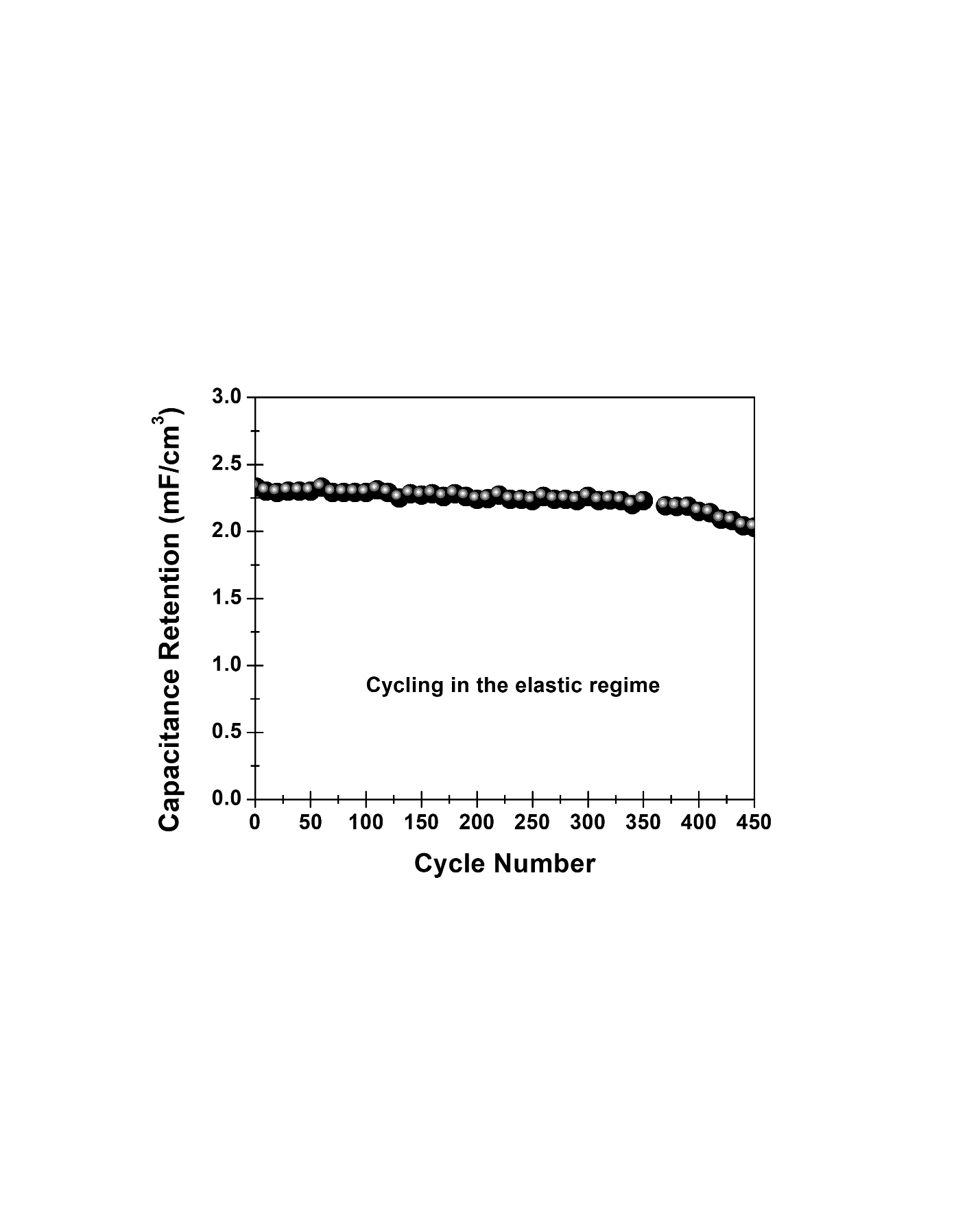


**Figure S19:** Capacitance retention of the supercapacitor after 450 charge/discharge cycles in the elastic regime.


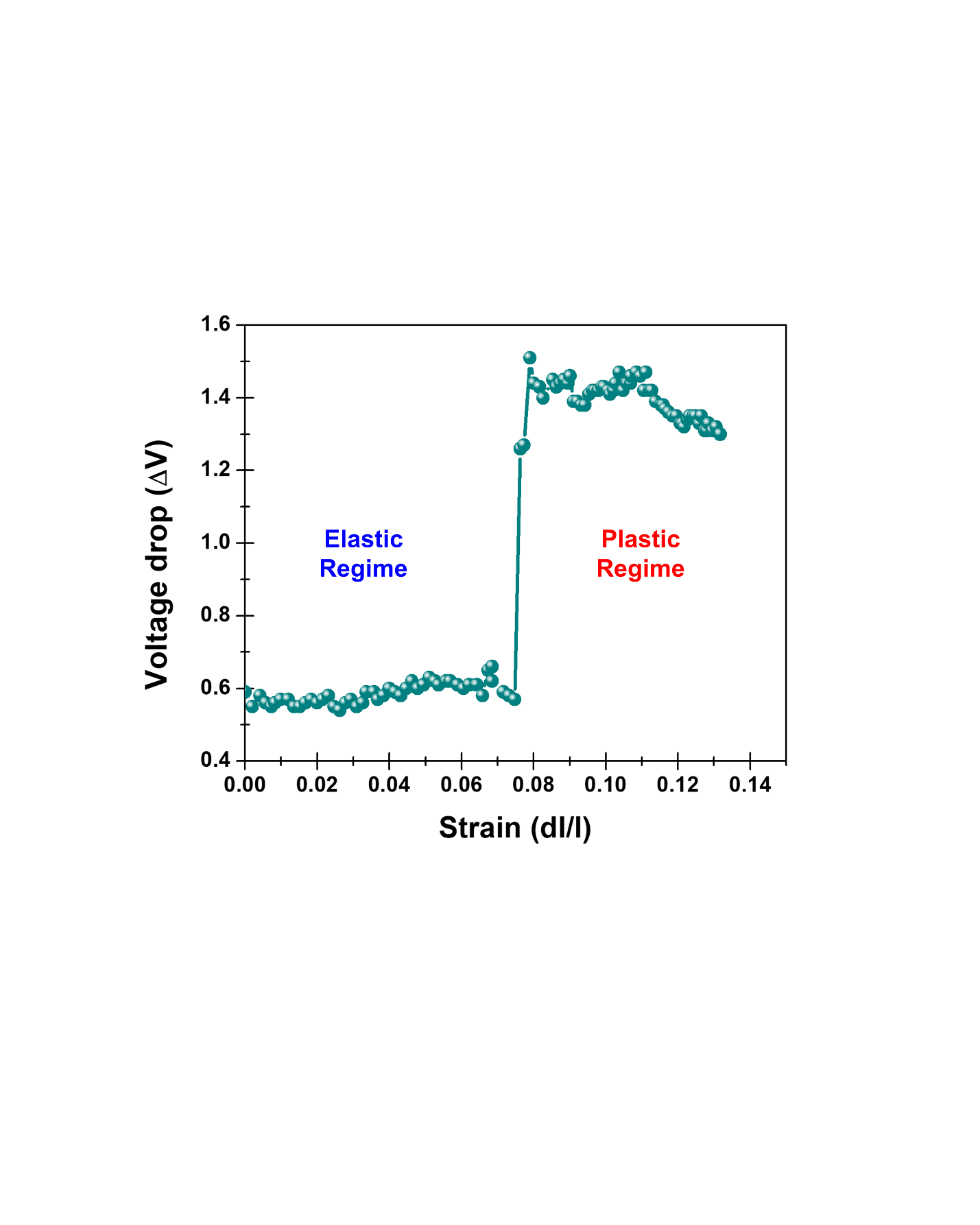


**Figure S20:** Voltage drop with increasing strain during in-situ mechano-electrochemical charge/discharge measurement of the multifunctional supercapacitor


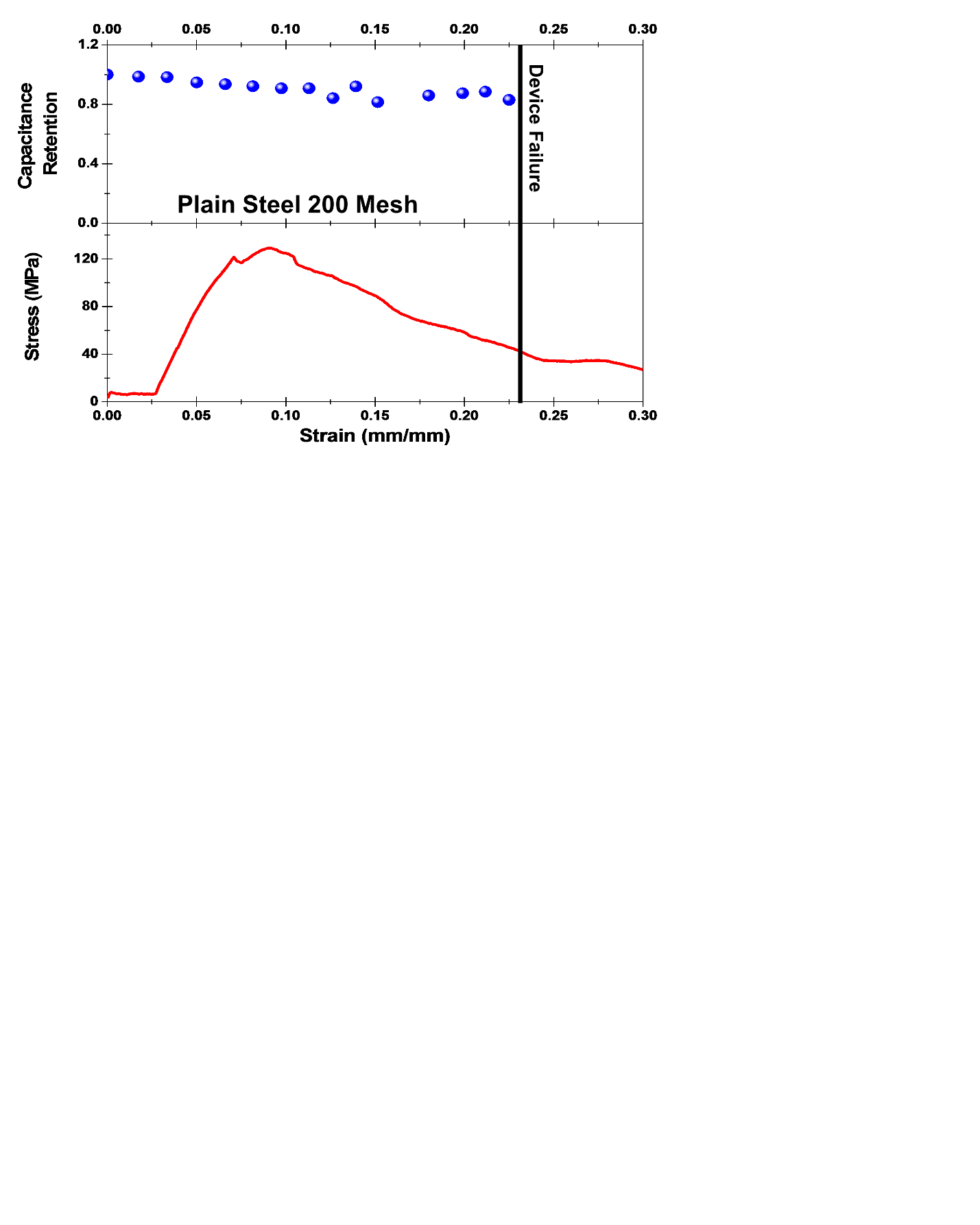


**Figure S21:** **In-situ mechano-electro-chemical tests.** In-situ mechano-electro-chemical tests of a SS/Kevlar/Epoxy-IL composite with the electrochemical performance on the top and the stress strain curve on the bottom.


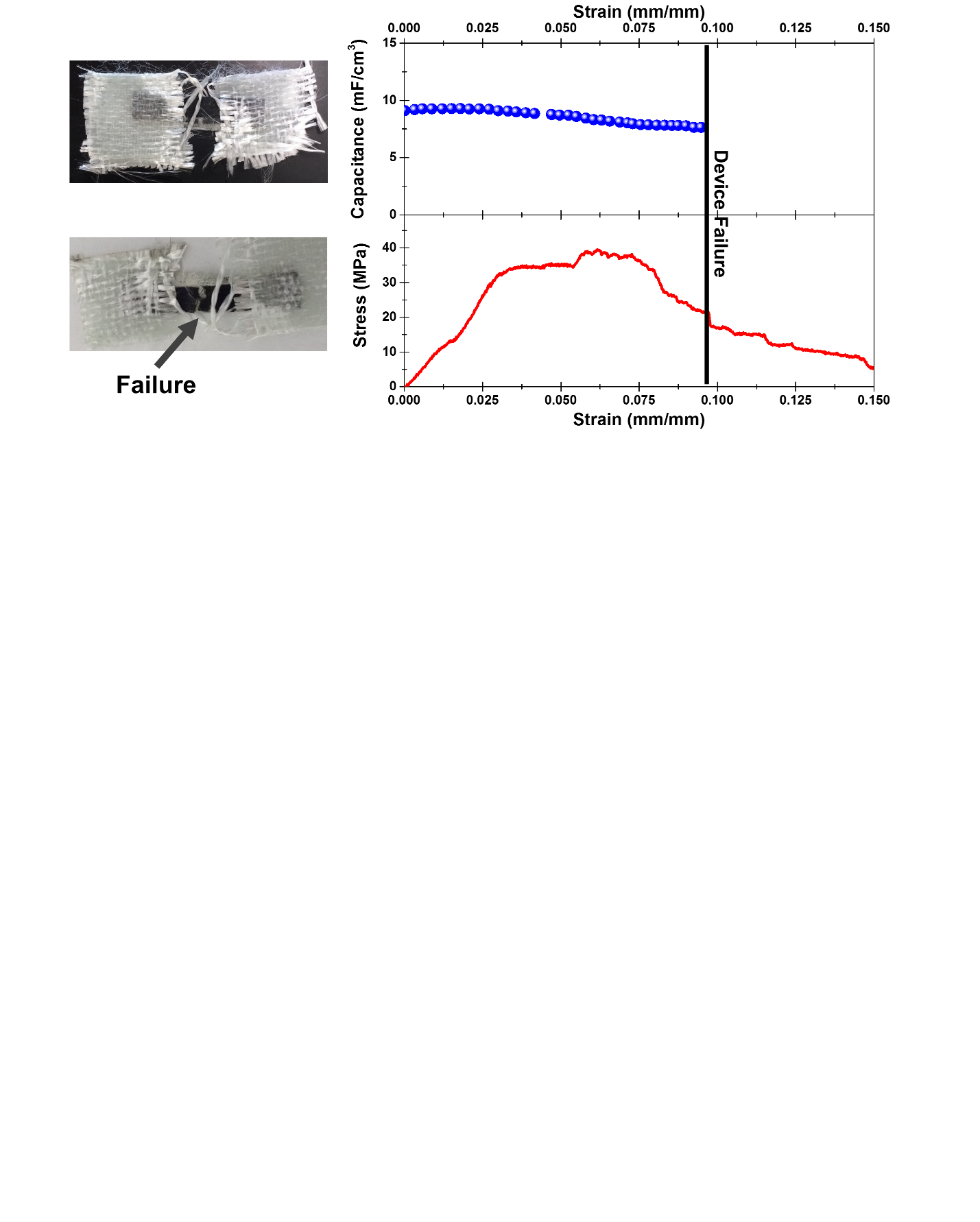


**Figure S22:** **In-situ mechano-electro-chemical tests.** On the left are the pictures of the devices before and after testing and on the right is the in-situ mechano-electro-chemical tests of a CNT-SS/Fiberglass/Epoxy-IL composite with the electrochemical performance on the top and the stress strain curve on the bottom.


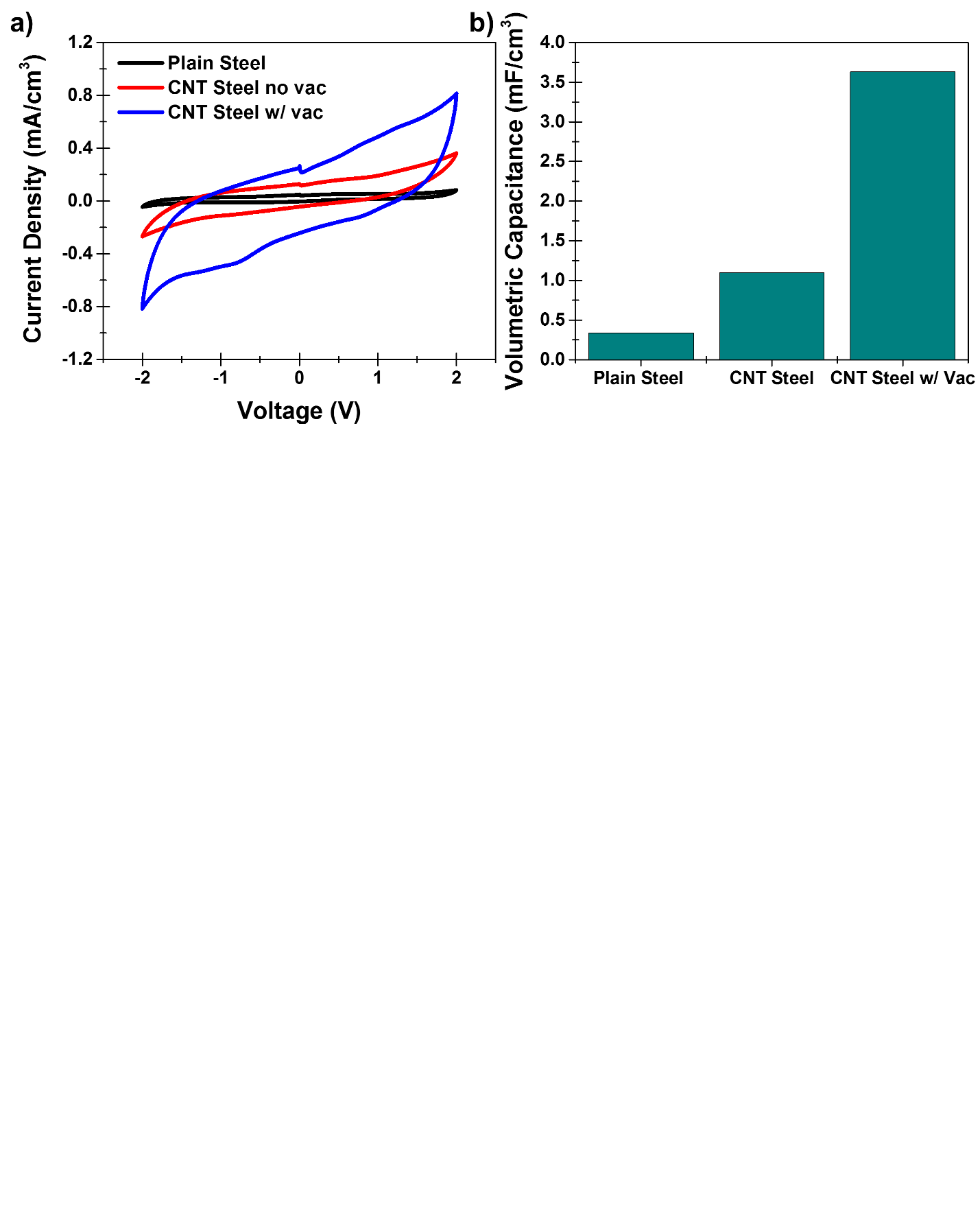


**Figure S23:** **Comparison of performance with and without vacuum infiltration.** a) CV curves of plain SS mesh (black), CNT grown on SS mesh without vacuum infiltration of the electrolyte (red), and CNT grown on SS mesh with vacuum infiltration of the electrolyte (blue). b) Comparison of the capacitance of the three devices in a).


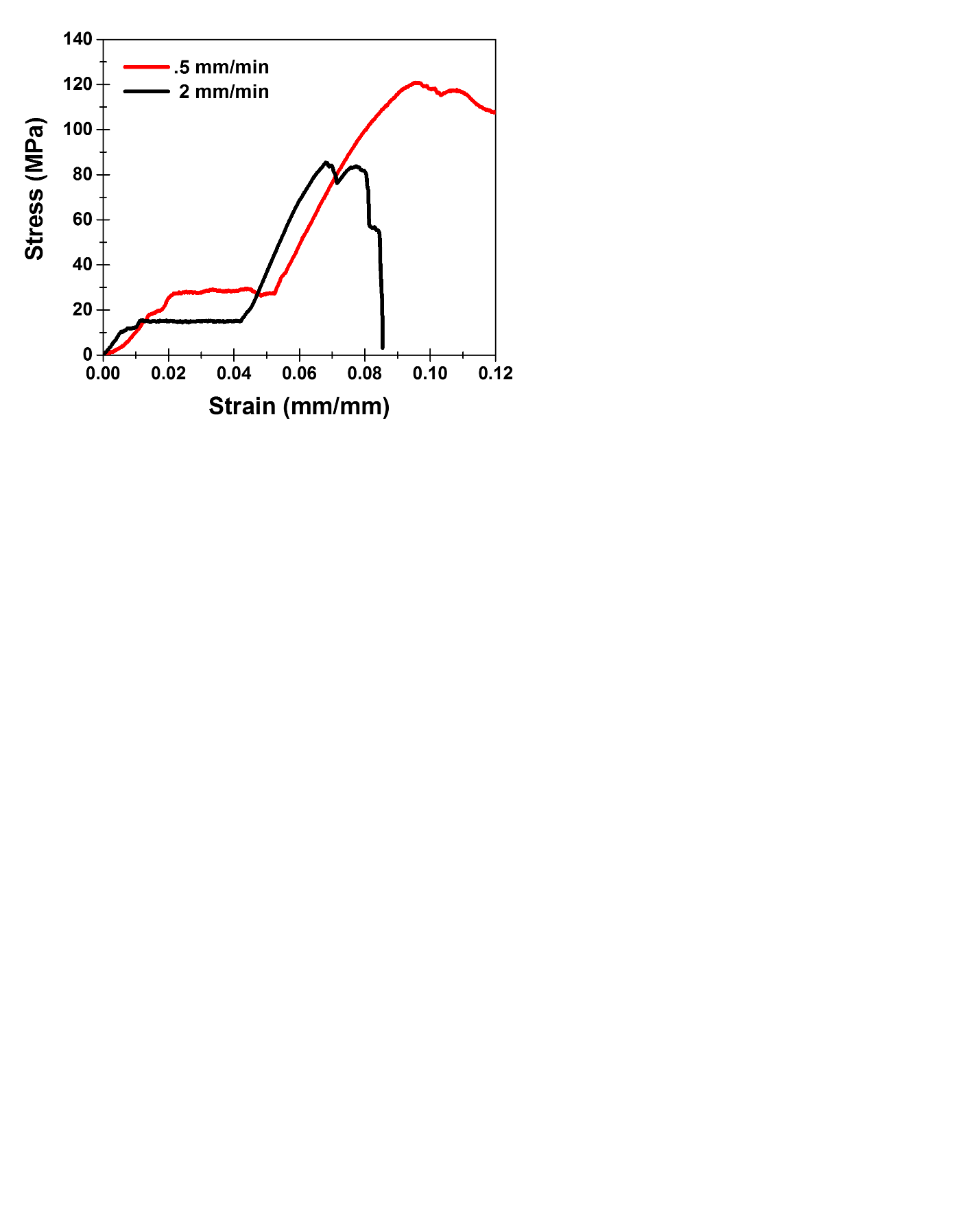


**Figure S24:** **Mechanical comparison of composites at different strain rates.**  Stress-strain curve comparing tensile tests of CNT-Steel/Kevlar/Epoxy-IL at different scan rates.

**References**

1 Karwa, M., Iqbal, Z. & Mitra, S. Scaled-up self-assembly of carbon nanotubes inside long stainless steel tubing. *Carbon* **44**, 1235-1242 (2006).

2 Teblum, E., Gofer, Y., Pint, C. L. & Nessim, G. D. Role of catalyst oxidation state in the growth of vertically aligned carbon nanotubes. *J. Phys. Chem. C* **116**, 24522-24528 (2012).

3 Qian, H., Kucernak, A. R., Greenhalgh, E. S., Bismarck, A. & Shaffer, M. S. Multifunctional structural supercapacitor composites based on carbon aerogel modified high performance carbon fiber fabric. *ACS Appl. Mater. Interfaces* **5**, 6113-6122 (2013).

4 Wang, Y., Qiao, X., Zhang, C. & Zhou, X. Development of All-Solid-State Structural Supercapacitor Using an Epoxy Based Adhesive Polymer Electrolyte. *ECS Transactions* **72**, 31-44 (2016).

5 Deka, B. K., Hazarika, A., Kim, J., Park, Y.-B. & Park, H. W. Multifunctional CuO nanowire embodied structural supercapacitor based on woven carbon fiber/ionic liquid–polyester resin. *Composites Part A: Applied Science and Manufacturing* **87**, 256-262 (2016).

6 Shirshova, N. *et al.* Structural composite supercapacitors. *Compos. Part A Appl. Sci. Manuf.* **46**, 96-107 (2013).

7 Senokos, E. *et al.* Energy storage in structural composites by introducing CNT fiber/polymer electrolyte interleaves. *Sci Rep.* **8**, 3407 (2018).
